# Transmasculine pregnancy—Occurrence, associations, and outcomes: A quantitative systematic review

**DOI:** 10.1111/aogs.70276

**Published:** 2026-07-08

**Authors:** Catherine Meads, Chelsea Daniels, Shawn Walker, Susan Bewley

**Affiliations:** ^1^ Faculty of Health, Medicine and Social Care Anglia Ruskin University Cambridge UK; ^2^ Faculty of Life Sciences & Medicine King's College London London UK; ^3^ Department of Women and Children's Health, School of Life Course Sciences King's College London London UK

**Keywords:** gravidity, non‐binary, obstetric, parity, pregnancy, trans man, transgender, transmasculine

## Abstract

**Introduction:**

Transmasculine people experience incongruence between their gender identity and sex (registered female at birth, AFAB). Little is known about transmasculine pregnancy. This project systematically reviews all published quantitative evidence on transmasculine pregnancy occurrence, associations, and outcomes, using midwifery, obstetric, and transgender health expertise.

**Material and Methods:**

Prospero protocol CRD 42020159034. Searches combined concepts of “transmasculine,” registered/assigned female at birth (AFAB), and “pregnancy,” using broad, inclusive sets of MeSH terms and keywords. Database searches: Embase, Maternity and Infant Care and Medline (OVID platform), and CINAHL (EBSCO platform) were searched from 2000 to August 2024, with no language restrictions. Included study references and relevant systematic reviews were also searched.

**Results:**

From 2166 citations, 44 studies were included, many of which were small and uncontrolled. Primary outcome: The mean proportion of transmasculine people ever having been pregnant (gravidity) was 5.6%–8.5% (47/835–321/3777, range 0%–16.3%), and the mean proportion having had births (parity) was 4.3%–8.5% (35/806–24/284, range 0%–19.1%). Secondary outcomes: The conception rates during testosterone use were low, but insufficient to rely upon as contraception; miscarriage rates appeared high (4/5 studies had rates between 31.6 and 40%—16/40 per person, 156/493 per pregnancy). Similarly, postnatal depression rates (two studies) range 15–58% (3/20–7/12). The limited data available suggested that testosterone use before pregnancy seemed to be associated with fewer conceptions compared to never having taken it, even where transmasculine people had stopped for several months before attempting conception. Pregnancy loss seemed to be higher in those exposed to testosterone antenatally. There was no or inadequate data on most other important outcomes, including access to care, assisted reproduction, stillbirths, and obstetric and neonatal complications.

**Conclusions:**

A significant minority of transmasculine people become pregnant, including after transition. The miscarriage findings warrant further investigation. Reproductive and maternity services should adapt, although minimal information risks negative consequences for users and providers, potentially adding to the lack of trust. Better data collection and research are urgently required in order to provide effective services.

## Graphical abstract

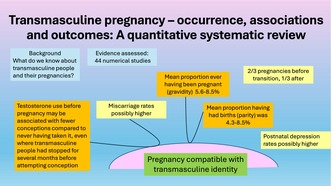

**Abbreviations**
AFAB/AMAB: registered (assigned) female/male at birth
ART: assisted reproduction techniques
IVF: in‐ vitro fertilization
PCOS: polycystic ovary syndrome
UK: United Kingdom
USA: United States of America

**Key message**

This quantitative systematic review of 44 studies shows that a significant minority of transmasculine people become pregnant, including after transition. Miscarriage and postnatal depression rates appeared high, but there was minimal information on most other important outcomes, demonstrating that more research is needed.

## 1 INTRODUCTION

Transgender people experience incongruence between their gender identity and birth sex.^1^ Trans men mostly identify as men, male or masculine, and non‐binary people as neither exclusively female nor male.^2^ Here, the term transmasculine denotes trans, non‐binary, and other gender expansive people registered (assigned) female at birth (AFAB). Non‐trans people are often referred to as cisgender. Research consistently shows that many transmasculine people are interested in pregnancy and parenting.^3^, ^4^ Alongside poor contraceptive knowledge,^5^, ^6^ unintended pregnancy and higher rates of sexual violence,^7^ intended transmasculine pregnancy is likely more common than some sensationalized reports suggest.^8^, ^9^

Some transmasculine people undergo transition—a process of changing various life aspects that may be social (e.g., living in a role), legal (e.g., name and/or gender/sex marker changes), medical (e.g., testosterone therapy), or surgical (e.g., mastectomy, hysterectomy +/− salpingo‐oophorectomy, metoidioplasty, phalloplasty). Some interventions compromise fertility.^10^ If a hysterectomy is performed as a prerequisite for sterilization for legal recognition^11^ or cancer prevention, pregnancy is precluded. Without these, many would elect to retain reproductive organs.^12^

Many transmasculine people use testosterone^13^ resulting in amenorrhea and changes to reproductive tract histology.^14^, ^15^, ^16^ Reversibility has been demonstrated observationally and experimentally,^17^ but little is known about its impact on timing or predictors of return to fertility, conception, pregnancy experiences, delivery, or post‐partum health. Despite internal and external pressures to undergo fertility‐compromising processes, the offer and uptake of fertility preservation is low, with high levels of decisional regret.^18^

Transmasculine people report historical and recent denial or difficulties when accessing reproductive healthcare.^19^ An early paper^20^ reported poor obstetric care, worsening dysphoria during pregnancy, mental health difficulties, and childhood adversity. More recent small US‐based studies found that most gynecologists^21^ and primary care physicians^22^ were willing to provide routine care, although 80% of obstetrician/gynecologists reported no training, with under a third feeling comfortable caring for transmasculine patients.^22^ Obstacles related to a lack of education^23^ and inadequate clinical guidance.^24^, ^25^

However, difficulty persists when accessing sexual and reproductive healthcare.^26^ There have been no reviews of the proportions of transmasculine people who become pregnant. Pregnancies may occur before, during, or after transition, which can take months or years^3^ and may include the use of testosterone and/or surgery, or not. Some also use assisted reproduction techniques (ARTs) via fertility clinics, including in vitro fertilization (IVF).

Current pre‐pregnancy guidance for transmasculine people wishing to become pregnant is precautionary, advising stopping testosterone as it may affect the baby's development^27^ and is teratogenic.^28^ The effects of continuing and higher exogenous testosterone levels in humans are unknown, as are obstetric outcomes.

Of seven recent systematic scoping reviews^29^, ^30^, ^31^ and systematic reviews^16^, ^19^, ^32^, ^33^ specific to transmasculine reproduction, three mostly focused on experiences of preconception and pregnancy care for transmasculine people,^19^, ^30^, ^31^ one did not combine and analyze studies,^29^ two focused on reproduction rather than obstetrics,^16^, ^32^ and one had an unclear number of included studies.^33^ These reviews reveal a mismatch between well‐documented experiences and sparse quantitative data. None provided summary estimates of gravidity, parity, or obstetric outcomes.

This systematic review determines the current state of knowledge of transmasculine pregnancy, including incidence, associations, and clinical outcomes. We estimate pregnancy rates in transmasculine people, whether pregnancies were associated with higher rates of reproductive and obstetric complications compared to cisgender women, the proportions taking testosterone during pregnancy, and any other reproductive and pregnancy‐related outcomes.

## 2 MATERIAL AND METHODS

This systematic review was conducted according to a prospectively registered protocol (PROSPERO; CRD42020159034). Qualitative outcomes and health service provider perspectives will be published separately.

### 2.1 Inclusion and exclusion criteria

Studies were eligible if they reported: (a) population: transmasculine people; (b) intervention/exposure: pregnancy; (c) comparators were not a pre‐requisite for study inclusion but differences between transmasculine and other participants were extracted; (d) one or more outcomes. The primary outcome was the incidence of transmasculine pregnancy, expressed as the proportion of a transmasculine sample/population. Secondary outcomes included miscarriage, termination, live and still birth, mode of delivery, birth parent, and/or neonatal complications. Primary qualitative, quantitative, and mixed‐methods studies were included with no restriction by language or study design, from 2000 onwards. Studies were eligible if they reported any aspect of trans pregnancy, for example, in participant characteristics tables, even if not the study focus.

Individual case reports, reviews, editorials, opinion pieces, letters, conference proceedings, book (chapters), unpublished reports, or any form of journal communication not classified as original research were excluded. No studies were excluded based on quality criteria.

### 2.2 Search strategy and study selection

A database search strategy was developed from previous systematic reviews, combining concepts of “transgender men and/or non‐binary persons AFAB” and “pregnancy” with broad and inclusive MeSH terms and keywords. This was supplemented by midwifery, obstetric, and transgender health expertise in the team, key experts on ResearchGate, review of references in included studies, and relevant systematic reviews. Embase (OVID), Maternity and Infant Care (OVID), Medline (OVID), and CINAHL (EBSCO) databases (platforms) were searched, with separate search strategies for the OVID and EBSCO platforms (Appendix S1, Tables S1 and S2). Searches were completed in August 2024.

Following de‐duplication, Microsoft Excel was used to order OVID and EBSCO results alphabetically and screen for duplicates. Titles and abstracts were screened independently by two reviewers against the eligibility criteria. The authors were contacted via email and/or ResearchGate if full texts were unavailable or to clarify queries. An independent full‐text assessment was carried out by two reviewers. Disagreements on inclusion, extracted data, or errors were resolved through team discussion.

### 2.3 Data extraction and quality assessment

Data extraction was completed independently by two reviewers, and all extracted data were collated. Participant descriptions used the terms provided in the original study. Study quality was assessed using methods‐appropriate Critical Appraisal Skills Programme checklists by one reviewer and checked by another, with disagreements resolved through discussion.

Quantitative data are presented as simple frequencies, percentages, and summary results, as reported in the included studies. Heterogeneity precluded statistical meta‐analysis. Studies were categorized into:

- Large cross‐sectional surveys comparing a relatively small sample of transmasculine people with a large sample of women, some in each group having been pregnant.
- Data collection of transmasculine people related to fertility, sexual or reproductive health, and pregnancy, with or without a comparator.
- Case series of other trans‐related topics (e.g., transitioning/hysterectomy) where pregnancy‐related data is incidental
- Studies where all participants are transmasculine people who have been pregnant or already delivered, with or without a comparator.

Each study type gives different kinds of information: A can give estimates of the proportions of transmasculine people who became pregnant or gave birth compared to women; B can give estimates of gravidity and parity (where gravidity numbers were not specific, parity was used instead); C can give gravidity and parity before medical transition; D can indicate the proportion of transmasculine people who gave birth before versus after transitioning. Studies with female comparators can also give a comparative prevalence of obstetric complications. Evidence about testosterone use as a contraceptive or during pregnancy, and obstetric complications, was also evaluated. Information needed to know the impact of transitioning on pregnancy rates, and obstetric/neonatal outcomes included comparisons of before versus after transitioning, having taken testosterone versus not, and bilateral mastectomy versus not.

### 2.4 Synthesis

The degree of heterogeneity of included study participants, settings, and outcomes reported made meta‐analysis impossible. Outcomes were not standardized across studies, and a wide variety of outcomes were reported. Findings were summarized narratively. Tables were used to highlight study characteristics, their differences, and any statistical test results. Raw percentages in each study were combined to give estimated gravidity and parity rates. As meta‐analysis was not possible, there was no assessment of reporting bias, generation of a funnel plot, or assessment of GRADE.^34^

## 3 RESULTS

From 2166 citations, 1397 original records were identified, 180 full‐text papers reviewed, and 44 published studies included—all in English. Figure S1 shows the PRISMA Flow Diagram. Five studies were reported in more than one paper, resulting in 51 papers with contributing evidence. Some of the additional papers reported a subgroup of the main study. Table S1 shows excluded studies and reasons.

### 3.1 Study characteristics

#### 3.1.1 Study design

A wide range of study types were included (Table S2): 3 type A,^35^, ^36^, ^37^ 14 type B,^6^, ^38^, ^39^, ^40^, ^41^, ^42^, ^43^, ^44^, ^45^, ^46^, ^47^, ^48^, ^49^, ^50^, ^51^, ^52^ 12 type C,^18^, ^53^, ^54^, ^55^, ^56^, ^57^, ^58^, ^59^, ^60^, ^61^, ^62^, ^63^, ^64^ 15 type D—4 with comparators (trans women,^65^ people registered/assigned male at birth (AMAB),^66^ cisgender women with either male or female partners,^67^ or heterosexual partnerships^68^) and 11 without.^69^, ^70^, ^71^, ^72^, ^73^, ^74^, ^75^, ^76^, ^77^, ^78^, ^79^, ^80^, ^81^, ^82^, ^83^

#### 3.1.2 Participants and setting

Transmasculine participants were described in a large variety of ways (Table S2). Results for transmasculine and transfeminine or gender non‐conforming (GNC) participants were clearly reported separately in seven studies.^35^, ^37^, ^39^, ^40^, ^46^, ^50^, ^51^, ^66^ In four, it was difficult to discern whether the text described participants of either/or both sexes or genders.^18^, ^44^, ^47^, ^48^, ^49^, ^65^ In one, the proportion of pregnant women was obtained via author correspondence.^18^

Participants were overwhelmingly recruited from the United States (*n* = 28 studies). Other countries/jurisdictions included Australia, Belgium, Brazil, Canada, China, Israel, the Netherlands, Saudi Arabia, Serbia, Sweden, and the European Union. Settings included: abortion facilities, gender clinics, hospitals, IVF clinics, LGBT health centers, national health systems, service user groups, support groups, and universities. Recruitment methods included consecutive series of clinic case notes, databases, flyers in social service agencies, snowball samples, and social media posts.

#### 3.1.3 Outcomes and quality

All included studies reported at least one numerical aspect of transmasculine pregnancy (Table S3). Study quality varied (Table S4), but almost all samples were too small to minimize the play of chance. Many reported pregnancy outcomes were obscure: for example, it was impossible to determine accurate numbers of transmasculine people,^50^, ^51^ or the proportion of participants giving birth (rather than partners) only being available in the supplementary material.^42^

### 3.2 Findings

#### 3.2.1 Prior diagnosis of polycystic ovary syndrome (PCOS)

Two studies reported relatively high PCOS rates pre‐pregnancy (Table 1).^64^, ^77^

**TABLE 1:** Associations and outcomes of pregnancy.

Findings/outcomes (chronological sequence)	Reference	Numbers (comparison where applicable)	Percentage (95% CI) and significance test	Study type
Before pregnancy/delivery				
PCOS before pregnancy	Light 2014	4/41 prior diagnosis of PCOS	9.80%	D
	Yaish 2021	27/56 had PCOS, 5 with frank phenotype	48.20%	C
Intentionality	Charlton	6/10 pregnant as teens	60%	D
		4/10 unintentional post teen	40%	
No prenatal care	Light 2014	2/41	4.90%	D
Pregnancy rates				
Currently pregnant	Tordoff 2019	Trans men (5/209)	2.40%	A
		vs. GNC adults (5/187)	2.70%	
		vs. cisgender women (2638/NR)	4%	
Prevalence ratio of pregnancy	Tordoff 2019	Trans men vs. cisgender women	0.38 (95% CI 0.13–1.15)	A
		Gender non‐conforming people vs. cisgender women	0.76 (95% CI 0.23–2.58)	
Ever pregnant (college students)	Reynolds 2021	Transmasculine (7/800)	0.90%	A
		vs. non‐binary AFAB (17/2236)	0.80%	
		vs. cisgender (1819/182622)	1.00%	
Total number of pregnancies	Light 2018	60 (from 32/197 participants)	1.85 per person	B
Early pregnancy losses				
Ectopic pregnancies	Cao 2021	1/48 (also 24/72 had unknown outcomes)	2.10%	C
	Moseson 2020, 2021, and 2022	2/433	0.50%	B
Pregnancy loss	Cao 2021	2/72 known to have had either a miscarriage or an abortion	2.70%	C
	Riggs 2020	16/51 participants had a pregnancy loss	31.40%	D
Miscarriages	Mattelin 2022	1/164 participants had a miscarriage	0.60%	B
	Charlton 2021	3/10 participants had spontaneous abortions	30%	D
	Ellis 2015	4/8 participants had at least one miscarriage	50%	D
	MacDonald x2	9/22 participants had miscarriages	36.40%	D
	Total mean miscarriage per participant	16/40	40.00%	D
	Light 2018	14/60 pregnancies were miscarriages (in 32 participants)	23.30%	B
	Moseson 2020, 2021, and 2022	142/433 pregnancies were miscarriages (in 210 participants)	33%	B
	Total mean miscarriage per pregnancy	156/493	31.60%	B
Abortions	Fix 2020	2/5	40%	B
	Gomez 2020	3/20 (accessed abortion services)	15%	B
	Mattelin 2022	1/164 (termination)	0.60%	B
	Moseson 2020, 2021, and 2022	67/1964	3.40%	B
	Total mean abortions per person	73/2153	3.40%	B
	Charlton	4/10 (induced abortions)	40%	D
	MacDonald 2016, 2021	2/22	9.10%	D
	Light 2018	9/60 total pregnancies were abortions	16.60%	B
Pregnancy outcomes				
Live birth	Mattelin 2022	3/146	2.10%	B
	Moseson 2020, 2021, and 2022	169/433	39.00%	B
	Charlton 2021	3/10	30%	D
	Leonard 2022	498/498	100%	D
	Van Amesfoort	5/5	100%	D
	Ghofranian 2022, 2023	5/5 (ART)	100%	B
	Leung 2019	2/2 (ART)	100%	B
Stillbirth	Moseson 2020, 2021, and 2022	2/171	1.20%	B
Proportions natural vs. IVF	Leung 2019	1/26 already had a child through natural conception	3.80%	B
Place of birth	Light 2014	Born in hospital (32/41)	78.00%	D
		At home (7/41)	17.10%	
		In independent birth centre (2/41)	4.90%	
Birth weight	Light 2014	3146 (SD 1671)	n/a	D
	MacDonald 2016, 2021	2722–4308 g	n/a	D
Gestational age at delivery	Light 2014	38 weeks (SD 6 days)	n/a	D
	MacDonald 2016, 2021	Range 36–42 weeks	n/a	D
Complication rates—mother				
Bleeding during pregnancy	Light 2018	6/32	18.80%	B
Anemia	Light 2014	3/41	7.30%	D
Gestational diabetes	Leonard 2022	Father giving birth (45/498)	9.10%	D
		vs. mother+mother (272/2572)	10.70%	
		vs. mother+father (147 638/1483119)	10.10%	
			(*p* = NS)	
	Light 2014	2/41	4.90%	D
	Stroumsa 2023	Transgender (at least 92/1902)	4.80%	D
		vs. cisgender (190 615/2721507)	vs. 7.0% (*p* = 0.03)	
Hypertension	Leonard 2022	Father giving birth (40/498)	8.00%	D
		vs. mother+mother (417/2572)	9.10%	
		vs. mother+father (135 405/1483119)	16.20%	
			(*p* = 0.001)	
	Light 2014	5/41	12.20%	D
	Stroumsa 2023	Transgender (180/1902)	9.50%	D
		vs. cisgender (369 135/2721507)	vs. 13.6%	
			(*p* = 0.17, *p* = 0.003)	
Low birthweight	Leonard 2022	Father giving birth (<12/498)	NR	D
		vs. mother+mother (80/2572)	3.40%	
		vs. mother+father (30 251/1483/119)	2.20%	
			(*p* = NR)	
Intrauterine growth restriction	Stroumsa 2023	Transgender (at least 11/1902)	0.60%	D
		vs. cisgender (27 231/2721507)	vs. 1.0%	
			(*p* = 0.48)	
Multiple pregnancy	Leonard 2022	Father giving birth (12/498)	2.40%	D
		vs. mother+mother (205/2572)	8.00%	
		vs. mother+father (22 342/1483/119)	1.50%	
			(*p* = 0.001)	
	Light 2014	2/41	4.90%	D
	Stroumsa 2023	Transgender (at least 57/1902)	3.00%	D
		vs. cisgender (53 766/2721507)	vs. 2.0%	
			(*p* = 0.001)	
Placental abruption	Light 2014	4/41	9.80%	D
Postpartum hemorrhage	Leonard 2022	Father giving birth (19/498)	3.80%	D
		vs. mother+mother (220/2572)	8.60%	
		vs. mother+father (65 810/1483119)	4.40%	
			(*p* = 0.001)	
Postpartum infection	Light 2014	2/41	4.90%	D
Preterm labor	Light 2014	4/41	9.80%	D
Preterm birth	Leonard 2022	Father giving birth (41/498)	8.30%	D
		vs. mother+mother (301/2572)	11.80%	
		vs. mother+father (112 093/1483119)	7.60%	
			(*p* = 0.001)	
	Stroumsa 2023	Transgender (166/1902)	8.70%	D
		vs. cisgender (266 621/2721507)	vs. 9.8%	
			(*p* = 0.44)	
Premature rupture of membranes	Light 2014	1/41	2.40%	D
Pyelonephritis	Light 2014	1/41	2.40%	D
Severe maternal morbidity	Leonard 2022	Father giving birth (<12/498)	NR	D
		vs. mother+mother (90/2572)	3.50%	
		vs. mother+father (25 287/1483119)	1.70%	
			(*p* = NR)	
	Stroumsa 2023	Transgender (at least 16/1902)	0.80%	D
		vs. cisgender (19 172/2721507)	vs. 0.7%	
			(*p* = 0.14)	
Uterine rupture	Light 2014	1/41	2.40%	D
Postnatal depression diagnosed	Falck 2024	7/12	58%	D
	MacDonald 2016	3/20	15.00%	D
Postnatal depression symptoms	MacDonald 2016	7/20	35.00%	D
	Pfeffer 2023	“a considerable number”/70	NR	D
Suicide attempts	Falck 2024	0/12	0%	D
Suicide ideation	Falck 2024	3/12	25.00%	D
Complication rates—baby				
Apgar score <7 at 5 mins	Leonard 2022	Father giving birth (<12/498)	NR	D
		vs. mother+mother (39/2572)	1.70%	
		vs. mother+father (12 182/1483119)	0.90%	
			(*p* = NR)	
Neonate admitted to the NICU	Light 2014	5/41	12.20%	D
Neonate diagnosed with an anomaly or developmental disorder	Light 2014	3/41 (1 each of ventricular septal defect, bone cancer, and sensory integration disorder)	7.30%	D
Neonate diagnosed with a disorder of sexual development	Light 2014	2/41 (intersex *n* = 1, micropenis *n* = 1)	4.90%	D

Abbreviations: NICU, neonatal intensive care unit; NR, not reported.

#### 3.2.2 Population pregnancy rates

No data on total transmasculine pregnancies or births at the end of reproductive life were found. Three type A studies that might have had such data reported other statistics. Currently, pregnant rates were 2% in trans men and 3% in gender non‐conforming adults versus 4% in cisgender women, with prevalence ratios of pregnancy in trans men and in gender non‐conforming people compared to cisgender women of 0.38 (95% CI 0.13–1.15) and 0.76 (95% CI 0.23–2.58), respectively.^37^ Previously, pregnancy rates in college students were 0.9% and 0.8% in transmasculine and non‐binary AFAB versus 1.0% in cisgender women.^36^ Proportions reporting that pregnancy interfered with their academic performance were 5.1% (of 237) transgender versus 0.6% (of 57 176) female students, although these were not pregnancy proportions, as the sample mixed AFAB and AMAB participants.^35^

#### 3.2.3 Gravidity and parity

The mean total gravidity and parity of transmasculine people in type B studies were at least 8.5% (range 3.4%–16.3%) and 8.5% (range 0%–19.1%), respectively, and in type C studies at least 5.6% (range 0%–12.1%) and 4.3% (range 0%–7.4%), respectively (Table 2). The average age of groups of individuals varied between 22.5 and 37.0 years.^63^, ^64^

**TABLE 2:** Gravidity and parity from type B and C studies.

Study name	Gravidity	Parity	Study type
Albar et al. (2023)	0% (0/18)	0% (0/18)	C
Baker et al. (2019)	—	2.9% (10/158)	C
Cao et al. (2021)	10.4% (5/48)	4.2% (2/48)	C
Cipres et al. (2017)	7.7% (2/26)	—	C
Grimstad et al. (2019)	—	19.1% (18/94)	B
Grimstad et al. (2023)	2% (1/50)	—	C
Hawkins et al. (2021)	—	7.4% (6/81)	C
Jeftovic et al. (2018)	—	1.6% (2/124)	C
Light et al. (2018)	16.3% (32/196)	—	B
Maheux et al. (2021)	3.4% (36/1053)	—	B
Malmquist et al. (2021)	—	0% (0/2)	B
Mattelin et al. (2022)	3.4% (5/164)	2.4% (4/164)	B
Moseson et al. (2020), Moseson et al. (2021), and Moseson et al. (2022)	10.7% (210/1964)	—	B
Obedin‐Maliver et al. (2017)	12.1% (4/33)	6.1% (2/33)	C
O'Hanlan et al. (2007)	—	4.9% (2/41)	C
Riggs and Bartholomaeus (2018)	6.4% (18/280)	—	B
Stark et al. (2019)	5.3% (8/150)	3.3% (5/150)	C
Vyas et al. (2021)	—	8.3% (2/24)	B
Wierckx et al. (2012)	—	6.0% (3/50)	C
Yaish et al. (2021)	—	2.9% (3/103)	C

Studies reporting proportions of parity with ART were 10.8% (5/46)^39^, ^40^ and 26.9% (7/26),^42^ with unclear numbers in two.^50^, ^52^ The average parity with ART from these few results was 16.7% (12/72). Table 1 shows individual studies' associations and pregnancy outcomes.

### 3.3 Impact of transitioning on becoming pregnant

#### 3.3.1 Contraception

Evidence of the contraceptive effectiveness of testosterone was sparse. One study reported that 36% (4/11) used testosterone as a contraceptive.^56^ Another reported that family planning advisors had told participants that pregnancy was unlikely while on testosterone,^6^ although one participant reported that his doctor had told him not to rely on it as a contraceptive. In one study, 16% (30/196) reported using testosterone as a contraceptive method, and 5.5% (10/196) reported that healthcare providers had advised this.^43^ In another, participants saw a tension between taking testosterone and getting pregnant.^62^ Two studies had qualitative comments on the use of testosterone as contraception.^6^, ^62^

#### 3.3.2 Relationship of transition to timing of pregnancy

One type B study reported the proportions of children to birth parents before and after transitioning of 60% (3/5) versus 40% (2/5).^50^ Two type D studies had quotes showing that pregnancies had occurred before and after transitioning, but with no summary data presented.^69^, ^75^ One reported about pregnancies before transitioning,^71^ and seven reported on pregnancies, probably described as after transitioning only.^67^, ^68^, ^70^, ^72^, ^76^, ^80^, ^81^, ^82^, ^83^

#### 3.3.3 Testosterone use and early pregnancy

Table 3 shows summary results of type D studies, one showed that 25% (4/12) never took testosterone prior to pregnancy, and 75% (8/12) did.^74^ Another reported that 22.7% (5/22) had never taken testosterone, 40.1% (9/22) had taken testosterone before conceiving, and 36.4% (8/22) only afterwards.^78^ Of type B studies, one found that 78% (38/49) reported pregnancies were in trans participants who had never taken testosterone, 20% in those who had and then stopped (10/49), and 2% (1/49) were taking it irregularly.^43^ A second reported that 3.5% (15/433) pregnancies occurred after initiating testosterone and 0.9% (4/433) while taking it.^47^ Half (2/4) of the latter miscarried (after 5 and 6 months of testosterone), one ended in abortion (4 months' testosterone); the last did not have testosterone duration or outcome reported. One study described that, of 5 transmasculine participants who gave birth, one each had testosterone, mastectomy, or both, and two had neither.^83^ Another described three trans men who had stopped taking testosterone (taken for 3–12 years), conceived spontaneously, and carried the pregnancies themselves.^64^

**TABLE 3:** Rates of pregnancy and testosterone use.

Study	On testosterone	Previous testosterone now stopped	Never on testosterone	Study type
Baker et al. (2019)	70% (7/10)	30% (3/10)		C
Falck et al. (2024)	NR	75% (8/12)	25% (4/12)	D
Light et al. (2018)	2.0% (1/49)	20.4% (10/49)	77.6% (38/49)	B
MacDonald et al. (2016)	NR	40.1% (9/22)	59.9% (13/22)	D
Moseson et al. (2020)	0.9% (4/433)	3.5% (15/433)	95.6% (414/433)	B
Van Amesfoort et al. (2023)	NR	40% (2/5)	60% (3/5)	D
Yaish et al. (2021)	NR	100% (3/3)	NR	C

#### 3.3.4 Mastectomy and pregnancy

Bilateral mastectomy rates in two studies were 50% (6/12) before pregnancy,^74^ and 40% (2/5) in transmasculine participants who had given birth.^83^

### 3.4 Early pregnancy losses

No comparative studies reported these outcomes. The ectopic pregnancy rate in one type B study was 0.5%^47^, ^48^, ^49^ and in one type C study was 2.1%.^55^ The fetal loss rate in one type C study^55^ was 2.7% and in one type D study^81^ was 31.4%. The miscarriage rate per person in one type B study^46^ was 0.6%, and the mean rate in three type D studies was 40% (Table 1). The total mean miscarriage per pregnancy in the two type B studies was 31.6%. The mean abortion rate per person in four type B studies was 3.4%. One type B study reported that 16.6% of total pregnancies were abortions.^43^ Another reported a 5% (1/20) need for access to abortion services.^52^ The abortion rate per person in type D studies varied between 9.1% and 40.0% (2 studies). Preferred modes of abortion in one study were 61.2% (41/67) surgical, 34.3% (23/67) medication, and 4.5% (3/67) other (primarily using herbs).^48^

### 3.5 Birth outcome and complications of pregnancy

Only one study reported the place of birth, with 23% (9/41) delivering out of hospital.^77^ Table 1 shows live birth rates reported in two type B studies that varied between 2.1% and 39.0%, and in three type D studies between 30% and 100%. Both studies reporting live birth rates following ART had 100% live birth rates.^39^, ^40^, ^42^ However, one reported combined rates in both transmasculine people and their female partners in the main text, but transmasculine only in a supplementary table.^42^ It also reported that 3.8% (1/26) transmasculine people had already delivered after natural conception.^42^ One study reported stillbirth—occurring in 0.5% of pregnancies, with a clinically, but not statistically, significant rate of 1.2% total births (2/171).^48^ Two studies reported gestational age at delivery (38 weeks (SD 6 days),^77^ range 36–42 weeks^78^, ^79^) and birth weights (mean 3146 g (SD 1671 g),^77^ range 2722–4308 g^78^, ^79^).

One type B and three type D studies reported a wide range of pregnancy complications (Table 1). Two were comparative^67^, ^68^: multiple pregnancy rates were similar in fathers giving birth versus mothers (with either mother or father partners) in one,^67^ but significantly higher in transgender versus cisgender participants in the other^68^; gestational diabetes was not significantly different in one^67^ but significantly lower in the other^68^; both reported lower rates of gestational hypertension for transmasculine participants; there were significantly lower rates of postpartum hemorrhage and preterm birth in one^67^ but no difference in preterm birth in the other,^68^ and no report of postpartum hemorrhage rates.

#### 3.5.1 Postnatal depression

Diagnosed postnatal depression rates varied between 15% (3/22)^79^ and 58.3% (7/12).^74^ The latter study found 16.7% (2/12) reported post‐partum psychotic symptoms and 25% (3/12) suicide ideation.^74^ A third study reported “[a] considerable”, but unspecified, number of 70 participants had postpartum depression.^80^

#### 3.5.2 Neonatal complications

One small study reported clinically significant rates of neonatal intensive care admission (12.2%, 5/41), anomaly or developmental disorders (7.3%, 3/41), and disorders of sexual development (4.9%, 2/41).^77^

#### 3.5.3 Cesarean section

Eleven studies compared cesarean and vaginal delivery rates (Table 4), with an estimated overall mean across study types B, C, and D of 25.4%. One comparative study^60^ found statistically significantly lower cesarean section rates in transgender men of 3.0% (1/33) versus cisgender women 16.8% (143/850) (*p* = 0.03).

**TABLE 4:** Cesarean section rates in transmasculine people.

Study	Cesarean sections	Vaginal deliveries	Known deliveries	Study type
Moseson et al. (2020), Moseson et al. (2021), and Moseson et al. (2022)	23% (39/169)	Presume 111/169	169/169	B
Cao et al. (2021)	40% (2/5)	Presume 3/5	5/72	C
Hawkins et al. (2021)	17% (1/6)	Presume 5/6	6/81	C
Obedin‐Malaver et al. (2017)	50% (1/2)	Presume 1/2	2/33	C
Stark et al. (2019)	20% (1/5)	4/5	5/5	C
Copeland et al. (2023)	50% (1/2)	Presume 1/2	2/2	D
Falck et al. (2021) and Falck et al. (2024)	25% (3/12)	9/12	12/12	D
Leonard et al. (2022)	28.9% (96/332)	236/332	332/498	D
Light et al. (2014)	29.3% (12/41) (25% of these elective)	29/41	41/41	D
Stroumsa et al. (2023)	21.1% (54/256)	202/256	256/256	D
Van Amesfoort (2023)	20% (2/5)	3/5	5/5	D
Total	25.4% (212/835)			

### 3.6 Impact of medical/surgical transition on pregnancy

#### 3.6.1 Chestfeeding/breastfeeding

One study reported that 20% (1/5) of chestfed their babies^83^; another that 41.5% (96/231) of trans men had exclusively chestfed, with 37.2% (86/231) chestfeeding for over 6 months.^66^ One study reported that 51.2% (21/41) of participants who became pregnant after transitioning had chestfed their babies—40% of those who had used pre‐pregnancy testosterone (10/25) versus 68.7% (11/16) who had not.^77^ Another reported that 72.7% (16/22) had chosen to chestfeed their babies, meaning some attempted chestfeeding despite having had chest masculinization surgery before pregnancy (40.9%, 9/22).^79^ Two participants with previous chest masculinization surgery experienced engorgement and early signs of mastitis for which they, and their health care providers, were ill‐prepared. Seven reported experiencing gender dysphoria while chestfeeding. Two study participants who had planned not to chestfeed, due to lack of chest tissue as well as anticipated gender dysphoria, reversed these decisions after birth.

#### 3.6.2 Impact of testosterone on outcomes

One small comparative study^77^ found no statistically significant differences in obstetric complications between participants with and without prior testosterone use (Table S5), except for chestfeeding/breastfeeding rate, which was higher without prior testosterone use.

## 4 DISCUSSION

From 2166 citations, 44 studies were included: 3 type A, 14 type B, 12 type C, and 15 type D. Included studies were heterogeneous: in terms of type, study design, countries, descriptions of transmasculine people, settings, recruitment strategies, and sizes. Although all contained some quantitative data regarding pregnancy, the quality varied. In a few, it was impossible to distinguish between transmasculine and transfeminine people.

Our systematic review, despite limited primary data, provides the best available evidence that a small but noteworthy minority of transmasculine people have pregnancies (~6%–9%) and children (~4%–9%). Type B study evidence was likely to have provided higher pregnancy rates because participants had a self‐selected interest in reproductive health, and type C studies may have provided underestimates, as the highest average age was 37.0 years.^63^ True values possibly lie between the estimates. Gravidity and parity would have been clearer had there been good‐quality type A studies reporting data for transmasculine people at the end of reproductive life. There were no data on proportions using ART and little evidence on its success.

There were several notable and concerning findings. The suggestion of a high miscarriage rate (~31%–40%) from several type D studies compares to ~11%–22% in the female population,^84^ but none of the studies reporting early pregnancy losses were comparative. Data about obstetric complications, including postnatal depression, were sparse. The two comparative studies^67^, ^68^ provided mixed results. Only one study reported baby complications, with a relatively high rate of diagnosed disorders of sexual development.^77^ The risk of mastitis was mentioned in one study,^79^ but rates are not known. Data were missing on access, late booking, avoidance of hospital care, or freebirthing, though one study reported that a quarter of births were outside the hospital.^77^

Approximately two thirds of transmasculine people's babies were born before transitioning and one third afterward, although definitions of populations and transitioning vary. Testosterone use before pregnancy seemed to be associated with fewer conceptions compared to never use, even where transmasculine people had stopped several months before attempting to conceive. Conception rates during testosterone use were low, but insufficient to rely upon as contraception. The small amount of data available suggests that pregnancy loss was higher in those who had already taken testosterone. Some transmasculine people were taking hormonal contraceptives (estrogen/progesterone or progesterone only) as well as testosterone, but there was no information about this combination on subsequent pregnancy. How these hormones interact is unknown. Some transmasculine people breastfed/chestfed their babies (range 20%–70%), including after chest masculinization surgery. Only one study reported rates of obstetric complications in those with prior testosterone use compared to those with no use.^77^ Although no statistically significant differences were reported, they did not report statistical checks on the combination of clinically significant lower gestational age and lower birthweights.

No previous systematic review on transmasculine pregnancy has investigated quantitative aspects or combined numerical pregnancy rate estimates. The finding of high postnatal depression rates is supported by a scoping review of transmasculine people's perinatal mental health, although that focused on qualitative themes of dysphoria, visibility, and isolation in transmasculine pregnancy,^30^ rather than postnatal depression rates. Their insights may indicate why our rates appear high and add validity.

Regarding strengths and limitations, our systematic review used a prespecified registered protocol, and inclusive wide‐ranging searches with no language restrictions, while confining itself to specific pregnancy topics. The extensive searching, double data entry, and analysis with discursive iteration and reflexivity from a diverse team add rigor. The definition of transmasculine was wide, capturing some studies with an apparently higher threshold for identity, such as registering as fathers, and others with non‐binary people who might not have revealed their gender identity to service providers. Quality appraisal was performed, although examining primary studies' overall methodological rigor may overestimate quality pertaining to this review's results; pregnancy data collected was often scanty and unrelated to individual studies' primary aims, generating simple counts, and percentages without denominators or comparators. Thus, insufficient primary studies may limit overall reliability and generalizability.

A noteworthy minority of transmasculine people become pregnant and have children—activities still compatible with their identity—which has patient implications in both gender‐affirming and maternity services. The extant literature suggests two thirds do so before transitioning, but demographic proportions and ages of transition may be changing.^85^ Although pregnancy rates in transmasculine people are low, the lack of reassuring outcomes information is a serious and important omission. If maternity services do not collect gender identity,^86^ lack of visibility and reliable information means that the reproductive impact of earlier medical interventions cannot be properly known and quantified. Fully informed consent for initial transitioning interventions is also imperiled if their impact on subsequent reproduction is unknown. Maternity staff cannot be aware of, nor offer, appropriate advice. Any potential deficits in care or perceived concerns may diminish trust, cause reduced access or avoidance,^31^ poorer attendance, more out‐of‐hospital births, and increase obstetric complications or postnatal problems. In addition, there are suggestions of more miscarriages and postnatal depression, but less hypertension. There is insufficient information to give reassurance about all other aspects. Maternity staff are less able to implement appropriate person‐centered care without useful data or education about transmasculine pregnancy.^23^ Counseling transmasculine people about gender‐affirming interventions during their reproductive years must include contraception and plans for possible childbearing, including the limitations of assisted reproduction. Testosterone cannot be relied upon for contraception. There is a need to create evidence‐based guidelines for use by multi‐disciplinary teams.

Service providers should be transparent about the current lack of knowledge and take the initiative to improve this through better quality research. More needs to be known about transmasculine people, their pregnancies, delivery, birth outcomes, and obstetric complications. Future research should concentrate on answering such questions alongside high‐quality care models. Reliable comparative data is urgently required, which could be collated by the UK Obstetric Surveillance System,^87^ given its national, prospective, case–control design for rare conditions (here, the combination of medical interventions and social complexity rather than obstetric pathology). Interviews with service users, healthcare professionals, and policy makers would triangulate findings. Data fields might include many missing factors of age, ethnicity, previous trauma, co‐morbidities, testosterone use, prescribed and recreational drugs, surgical history, and antenatal care. Recruitment would be required from maternity units, emergency gynecology, and abortion services. Given high rates of prior trauma,^88^ distrust and “going by stealth,”^31^ novel community recruitment could be tested with stakeholder involvement from the outset in design, interpretation, and dissemination, as per the Survivors' Voices research ladder of participation.^89^ A James Lind Alliance Initiative has already identified service users' research priorities (I. Levene, unpublished data). Rates of reproductive regret regarding infant feeding after younger age mastectomy, barriers to maltreatment, and facilitators for better care all need investigation. Guidance about definitions and data collection will help researchers contend with changing language and provide clearer categories.

## 5 CONCLUSION

This systematic review of 44 studies shows that a significant minority of transmasculine people become pregnant, including after transition. Of these pregnancies, there were several notable and concerning findings, but there was no or inadequate data on many important outcomes. Conception rates during testosterone use were low, but insufficient to rely upon as contraception. The apparently higher miscarriage rates warrant further investigation. Pregnancy loss seemed to be higher in those exposed to testosterone antenatally. Reproductive and maternity services should adapt, although minimal information risks negative consequences for users and providers, potentially adding to a lack of trust. Better data collection and research are urgently required in order to provide effective services.

## AUTHOR CONTRIBUTIONS

Susan Bewley had the original idea for the work and led the team in registering the protocol on PROSPERO. Susan Bewley, Shawn Walker, and two collaborators were involved in planning the original project, and Susan Bewley wrote an initial draft. Catherine Meads conducted the searches. Chelsea Daniels and Catherine Meads checked citations and extracted data. Catherine Meads conducted a risk of bias assessment. Catherine Meads wrote the final version, which was edited by all authors. The authors accept responsibility for the final version of the paper as published.

## FUNDING INFORMATION

The authors have nothing to report.

## CONFLICT OF INTEREST STATEMENT

No financial interests. SB was the founder co‐chair of GLADD (the UK‐based association for LGBT doctors and dentists), and her interests can be found at https://www.whopaysthisdoctor/58.

## Supporting information


Appendix S1.
**Figure S1**.
**Table S1**.
**Table S2**.
**Table S3**.
**Table S4**.
**Table S5**.

## Data Availability

Data sharing not applicable to this article as no datasets were generated or analysed during the current study.
