# Supplementary material for: Transmasculine pregnancy—Occurrence, associations, and outcomes: A quantitative systematic review
Source: Acta Obstet Gynecol Scand. 2026 Jul 8;105(9):1646–61. doi: 10.1111/aogs.70276 (PMC13394970; doi:10.1111/aogs.70276)
Supplement: Supplementary file 1 [file AOGS-105-1646-s001.docx]

Supplementary Material

**Transmasculine pregnancy – occurrence, associations and outcomes: a quantitative systematic review**

Appendix S1. Example search strategy

Figure S1. Prisma diagram

Table S1. List of excluded studies with reasons for exclusion

Table S2. Characteristics of included studies

Table S3. Quantitative results

Table S4. Quality assessment using CASP questionnaire

Table S5. Obstetric complications in participants with prior testosterone compared to none (from Light et al., 2014)

Appendix S1 Example search strategy

Database: Embase <1974 to 2024 July 15>

--------------------------------------------------------------------------------

1	transgender.mp. or female to male transgender/ or "transgender and gender nonbinary"/ or transgender/ (20841)

2	non-binary gender.mp. (132)

3	non binary gender.mp. (132)

4	gender non-conforming.mp. or gender nonbinary/ (734)

5	gender nonconforming.mp. (698)

6	gender fluid.mp. (84)

7	gender queer.mp. (61)

8	trans man.mp. (57)

9	transman.mp. (41)

10	trans male.mp. (98)

11	transmasculine.mp. (549)

12	trans masculine.mp. (95)

13	transmasc*.mp. (554)

14	transgenderism/ (427)

15	1 or 2 or 3 or 4 or 5 or 6 or 7 or 8 or 9 or 10 or 11 or 12 or 13 or 14 (21452)

16	pregnancy.mp. or multiple pregnancy/ or pregnancy rate/ or pregnancy/ (1055671)

17	peripartum period.mp. (2031)

18	postpartum period.mp. (16461)

19	pregnancy trimesters.mp. (310)

20	gravidity.mp. (4931)

21	obstetric labor.mp. or labor/ (28053)

22	obstetric labour.mp. (92)

23	caesarean section.mp. or cesarean section/ (136577)

24	vaginal birth.mp. or vaginal delivery/ (49124)

25	obstetric surgical procedures.mp. (67)

26	cervical cerclage.mp. or uterine cervix cerclage/ (3156)

27	obstetric delivery.mp. or obstetric delivery/ (24262)

28	episiotomy.mp. or episiotomy/ (6548)

29	maternal age.mp. or maternal age/ (58880)

30	maternal-fetal exchange.mp. (258)

31	fetal.mp. (381008)

32	fertility/ or fertility.mp. (173128)

33	parity.mp. or parity/ (67438)

34	parturition.mp. or birth/ (38040)

35	placentation.mp. or placenta development/ (12540)

36	gestation.mp. (202224)

37	gestational age.mp. or gestational age/ (227964)

38	gestational weight gain.mp. or gestational weight gain/ (7940)

39	lactation.mp. or lactation/ (77401)

40	breast feeding.mp. or breast feeding/ (75536)

41	abortion/ or spontaneous abortion/ or abortion.mp. (136535)

42	ectopic pregnancy.mp. or ectopic pregnancy/ (24098)

43	Apgar score/ or apgar.mp. (42719)

44	birth weight.mp. or birth weight/ (165284)

45	small for date infant/ or small for dates baby.mp. (19163)

46	large for gestational age/ (5283)

47	newborn disease.mp. or newborn disease/ (24660)

48	pregnan*.mp. (1157279)

49	obstetric.mp. (120584)

50	chestfeeding.mp. (43)

51	termination of pregnancy.mp. or pregnancy termination/ (20614)

52	miscarriage.mp. (27034)

53	small for gestational age.mp. or small for gestational age/ (20494)

54	16 or 17 or 18 or 19 or 20 or 21 or 22 or 23 or 24 or 25 or 26 or 27 or 28 or 29 or 30 or 31 or 32 or 33 or 34 or 35 or 36 or 37 or 38 or 39 or 40 or 41 or 42 or 43 or 44 or 45 or 46 or 47 or 48 or 49 or 50 or 51 or 52 or 53 (1821342)

55	15 and 54 (1547)

Figure S1 Prisma diagram for transmasculine pregnancy systematic review (no automation tools used)

**Identification of studies via other methods**

**Identification of studies via databases and registers**

Records identified from:

Websites (n = 13)

Records removed *before screening*:

Duplicate records removed (n = 769)

Records identified from:

Databases (n = 2153)

Embase = 1401

Medline = 595

CINAHL = 146

MIDIRS = 11

Registers (n = 0)

**Identification**

Records screened

(n = 1384)

Records excluded

(n = 1217)

Reports not retrieved

(n = 0)

Reports sought for retrieval

(n = 13)

Reports sought for retrieval

(n = 167)

Reports not retrieved

(n = 0)

**Screening**

Reports excluded: 13

Unpublished report (n = 4)

Not about pregnancy (n=9)

Reports excluded: 116

No pregnancy data (n = 42)

Review/editorial (n=24)

Conference abstract (n = 19)

Not transmasculine (n=9)

Healthcare workers (n=8)

Case report (n = 8)

Other (n=6)

Reports assessed for eligibility

(n = 13)

Reports assessed for eligibility

(n = 167)

Studies included in review

(n = 44)

Reports of included studies

(n = 51)

**Included**

Table S1. List of excluded studies with reasons for exclusion

| **No** | **Reference** | **Reason** |
| --- | --- | --- |
| 1 | Adams E. If transmen can have babies, how will perinatal nursing adapt? *The American Journal of Maternal Child Nursing*, 2010;*35*(1), 26–32. [https://doi.org/10.1097/01.NMC.0000366807.67455.DE](https://protect.checkpoint.com/v2/r02/___https://doi.org/10.1097/01.NMC.0000366807.67455.DE___.YzJlOmFuZ2xpYXJ1c2tpbnVuaXZlcnNpdHk6YzpvOmNmYWYxNDhkYTExYzQ2N2NkYmJjYTNmZjdjNTY5ZWU2Ojc6OTZmNzo5NjNkZWUxN2ZmZGFjNGRiOTZlZWMzNWY5ZjE1MWQ0NjQwYTI1NWYxYTY0ZmIyZTQwZjU1NmQwNjk1MzEzNTQ0OnA6VDpG) | Single case report |
| 2 | Adeleye A, Cedars M, Smith J, et al. Ovarian stimulation for fertility preservation or family building in a cohort of transgender men. *Journal of Assisted Reproduction and Genetics*, 2019;*36*(10), 2155–2161. [https://doi.org/10.1007/S10815-019-01558-Y](https://protect.checkpoint.com/v2/r02/___https://doi.org/10.1007/S10815-019-01558-Y___.YzJlOmFuZ2xpYXJ1c2tpbnVuaXZlcnNpdHk6YzpvOmNmYWYxNDhkYTExYzQ2N2NkYmJjYTNmZjdjNTY5ZWU2Ojc6NGQ1OTpiNGU5MjY5N2E3ZTFiY2QxODc1Y2U0NDhkNzU1YjZlZDBjYmJjZWFiNWE0NTQ0ZWMyMzRhODNlNmQyY2JhYTgzOnA6VDpG) | No reports of pregnancy among participants |
| 3 | Agénor M, Cottrill AA, Kay E, et al. Contraceptive beliefs, decision making and care experiences among transmasculine young adults: A qualitative analysis. *Perspectives on Sexual and Reproductive Health*, 2020;*52*(1), 7–14. [https://doi.org/10.1363/PSRH.12128](https://protect.checkpoint.com/v2/r02/___https://doi.org/10.1363/PSRH.12128___.YzJlOmFuZ2xpYXJ1c2tpbnVuaXZlcnNpdHk6YzpvOmNmYWYxNDhkYTExYzQ2N2NkYmJjYTNmZjdjNTY5ZWU2Ojc6ZTJiMDo0NmYwYTc2NGY0YmVkMjEzMzY4YmIxMGU0MGFiZWI4YjEwMzQzZDQwMGNlNjRkODVhOTFhMTk5MTQ5ZGZmNWI4OnA6VDpG) | Focus is contraception, not pregnancy |
| 4 | Agenor M, Murchison GR, Najarro J et al. Mapping the scientific literature on reproductive health among transgender and gender diverse people: a scoping review. *Sexual and Reproductive Health Matters* 2021;29(1):57-74, | Review |
| 5 | Allen CL, Muschialli L, Nihlen A, et al. Barriers to sexual and reproductive health care faced by transgender and gender diverse people: a systematic review. *Reproductive Health* 2025;22:115 | Review |
| 6 | Almvik Å, Ostad H, Dahl B. Being a man, but using the body as a woman - Norwegian midwives’ thoughts on providing antenatal care for pregnant transmen. *Sexual & Reproductive Healthcare,* 2023;35:100822. | Healthcare workers only |
| 7 | Araya A, Shumer D, Warwick R et al. “I think sex is different for everybody” – Sexual experiences and expectations in transgender youth. *Journal of Pediatric and Adolescent Gynecology* 2023;33(2):209-210 | Conference abstract |
| 8 | Arif I, Dahlen S, Connolly D et al. Obstetrics & Gynaecology in International Clinical Practice Guidelines for Gender Minority/Trans People. BJOG 2021;2090 | Conference abstract |
| 9 | Asklöv K, Ekenger R, Berterö C. Transmasculine persons’ experiences of encounters with health care professionals within reproductive, perinatal, and sexual health in Sweden: A qualitative interview study. *Transgender Health*. 2021;2;6(6):325-331 [https://doi.org/10.1089/TRGH.2020.0081](https://protect.checkpoint.com/v2/r02/___https://doi.org/10.1089/TRGH.2020.0081___.YzJlOmFuZ2xpYXJ1c2tpbnVuaXZlcnNpdHk6YzpvOjFlOGYzOGVjNDYzZjBiODQwNDgyZTBmNjU2NGU0NGQ4Ojc6YWJlZDoxNDMzOWMyMThjNzBhNDA0NjAxMzdiYzkzYjhmZTZiMmQ0YjJmNGRkMGMyMWU4MGY2ZGJkMmZiNTkyNjBmODE3OnA6VDpG) | No data specific to pregnancy |
| 10 | Bargallo MF, Galdu M, Juanmarti SG et al. Artificial reproductive technology in transgender men and cisgender women: does testosterone administration before ovarian stimulation have an impact on IVF embryological and clinical outcomes? *Fertility and Sterility* 2023;120(4):Supp e16 | Conference abstract |
| 11 | Bartels CB, Uliasz TZ, Lestz L et al. 2021 Short-term testosterone use in female mice does not impair fertilizability of eggs: implications for the fertility care of transgender males. Human Reproduction 2021;36(1):189-198. | Study done on mice |
| 12 | Batham S, Pilkington V, Brady M. The contraceptive needs of transgender men and non-binary people. *Sexually Transmitted Infections* 2023;P009 | Nothing about pregnancy |
| 13 | Berger AP, Carver LA, Potter EM, et al. Pregnant transmen and barriers to high quality healthcare - the trans* community defining the trans* population. *Proceedings in Obstetrics and Gynecology*, 2015;*5*(2), 1–13. [http://ir.uiowa.edu/pog/](https://protect.checkpoint.com/v2/r02/___http://ir.uiowa.edu/pog/___.YzJlOmFuZ2xpYXJ1c2tpbnVuaXZlcnNpdHk6YzpvOmNmYWYxNDhkYTExYzQ2N2NkYmJjYTNmZjdjNTY5ZWU2Ojc6MzVhNzo0MjczZTBjZDU3YWI3YjEzNTMxNmJiNjZmMzQ0YWYyZDc1ZmIxYWQ2ZjFjOGQwNzAzZjdmMmI5OTQzNWU3NWNiOnA6VDpG) | Review |
| 14 | Botelle R, Connolly D, Walker S, et al. Contemporary and future transmasculine pregnancy and postnatal care in the UK. *The Practising Midwife* 2021;24(5):1-7 | Commentary article with no original data |
| 15 | Bower-Brown S. Trans and/or non-binary (TNB) parents’ experiences during the perinatal period. *Human Reproduction* 2023;37(1):P502 | Conference abstract |
| 16 | Bower-Brown S, Zadeh S. “I guess the trans identity goes with other minority identities”: An intersectional exploration of the experiences of trans and non-binary parents living in the UK. *International Journal of Transgender Health* 2021;22(1–2):101–112 | Not specific to transmasculine people |
| 17 | Bowler S;Vallury K ;Sofija E. Understanding the experiences and needs of LGBTIQA+ individuals when accessing abortion care and pregnancy options counselling: a scoping review. *BMJ Sexual and Reproductive Health* 2023;49(3):192-200 | Review |
| 18 | Bozlack CT, Ruland L, Eskew B et al. Breastfeeding disparities and recommended strategies to end them in New York. Preventive Medicine Reports 2024;47:102881 | Not specific to trans men, |
| 19 | Bozlak C, Ruland L, Eskew B, et al. Addressing breastfeeding disparities in New York State: Findings from a 2021 qualitative study. *Current Developments in Nutrition* 2023;7:101069 | Nothing about pregnancy |
| 20 | Brandt JS, Patel AJ, Marshall I, et al.Transgender men, pregnancy, and the “new” advanced paternal age: A review of the literature. *Maturitas* 2019;128:17–21 | Review |
| 21 | Carroll R, Sepulveda B, McLeod L, et al. Characteristics and gender affirming healthcare needs of transgender and non-binary students starting hormone therapy in a student health service in Aotearoa New Zealand. *Journal of Primary Health Care.* 2023 Jun;15(2):106-111. | No participants with an obstetric history |
| 22 | Charter R, Ussher JM, Perz J, et al. The transgender parent: Experiences and constructions of pregnancy and parenthood for transgender men in Australia. *International Journal of Transgenderism*. 2018;19(1):64–77. | Qualitative results only |
| 23 | Chiland C, Clouet AM, Golse B, et al. A new type of family: Transmen as fathers thanks to donor sperm insemination. A 12-year follow-up exploratory study of their children. *Neuropsychiatrie de l’Enfance et de l’Adolescence*, 2013;*61*(6), 365–370. [https://doi.org/10.1016/J.NEURENF.2013.07.001](https://protect.checkpoint.com/v2/r02/___https://doi.org/10.1016/J.NEURENF.2013.07.001___.YzJlOmFuZ2xpYXJ1c2tpbnVuaXZlcnNpdHk6YzpvOmNmYWYxNDhkYTExYzQ2N2NkYmJjYTNmZjdjNTY5ZWU2Ojc6NTY1YjozMDNmZDI3OWJhNDdkYjRkNzZiZjY1YWRjNzlmMDExMWFhN2UwODc4Zjc3Nzc2Yjg1MTQxODNkNWUwMzRmODExOnA6VDpG) | Trans participants were not gestational parents |
| 24 | Chiu DW, Jones RK. Sexual orientation among a national sample of people obtaining abortions in 2021-2022. *Contraception* 2022;116:84 | Conference abstract |
| 25 | Chu H, Kirby L, Booth A, et al. Providing gender affirming and inclusive care to transgender men experiencing pregnancy. *Midwifery* 2023;116:103550. | Healthcare workers only |
| 26 | Conard RS, Folsom LJ. Assessment of family planning readiness in transgender youth. *Journal of the Endocrine Society* 2023; 5;7(Suppl 1):bvad114.2079 | Conference abstract |
| 27 | Eiduson R, Murchison GR, Agénor M, et al. Sexual healthcare experiences of nonbinary young adults. *Culture, health & sexuality*, 2021;24(10):1319-1335 | No mention of pregnancy |
| 28 | Factor RJ, Rothblum ED. A study of transgender adults and their non-transgender siblings on demographic characteristics, social support, and experiences of violence. Journal of LGBT Health Research, 2007;3(3), 11-30. | No data on pregnant trans men |
| 29 | Florensa M, Galdu M, Penarrubia J et al. Fertility treatments among the transgender community: demographics, type of treatments and clinical outcomes. Fertility and Sterility 2023;120(4):Supp E159 | No data on pregnant trans men |
| 30 | Gedzyk-Nieman SA, McMillian-Bohler J. Inclusive care for birthing transgender men: a review of the literature. *Journal of Midwifery and Women's Health* 2022;67(5):561-568. | Review |
| 31 | Ghofranian A, Aharon D, Friedenthal J et al. Supporting transgender male patients through fertility treatment: Aiding creation of the modern family through assisted reproductive technology. *Fertility and Sterility* 2021;116(3):Supp E271 | Conference abstract |
| 32 | Ghofranian A, Estevez SL, Gellman C et al. Assisted reproductive technology treatment outcomes in transgender males with history of gender affirming hormone therapy. *Fertility and Sterility* 2022:118(4):Supp E21-22 | Conference abstract |
| 33 | Glaser R, Newman M, Parsons M, et al. Poster presentation report: Safety of maternal testosterone therapy during breast feeding. *The International Journal of Pharmaceutical Compounding*, 2009;*13*(4),314–317. [https://ijpc.com/Abstracts/Abstract.cfm?ABS=2966](https://protect.checkpoint.com/v2/r02/___https://ijpc.com/Abstracts/Abstract.cfm?ABS=2966___.YzJlOmFuZ2xpYXJ1c2tpbnVuaXZlcnNpdHk6YzpvOmNmYWYxNDhkYTExYzQ2N2NkYmJjYTNmZjdjNTY5ZWU2Ojc6MGZjMjowM2UyOWZhYTNjMDk3NGJjZTBhZGQ0MGY3Y2E0YTEzMzM0Y2EwNTFmNTRlZTE0M2NhZWM3OGU2YTVkYjhhYzdjOnA6VDpG) | Conference abstract |
| 34 | Gomez AM, Bennett AH, Arcara J et al. Estimates of use of preferred contraceptive method in the United States: a population-based study. *The Lancet Regional Health Americas*. 2024;30:100662 | No obstetric data from trans men |
| 35 | Greenfield M, Darwin Z. Trans and non-binary pregnancy, traumatic birth and perinatal mental health: a scoping review. *International Journal of Transgender Health* 2021;22(1–2):203–216 | Review |
| 36 | Hallstrom S, Grundstrom H, Malmquist A, et al. Fear of childbirth and mental health among lesbian, bisexual, transgender and queer people: a cross-sectional study. *Journal of Psychosomatic Obstetrics and Gynecology* 2022;43(4):526-531 | Not specific to transgender people |
| 37 | Harris JK, Hernandez O. Measurement of sex and gender identity in the Behavioural Risk Factor Surveillance System Survey, 2014 to 2020. *Journal of Health Care in the Poor & Underserved* 2023;34(2):585-597 | No data on pregnant trans men |
| 38 | Hoffkling A, Obedin-Maliver J, Sevelius J. From erasure to opportunity: a qualitative study of the experiences of transgender men around pregnancy and recommendations for providers. *BMC Pregnancy Childbirth.* 2017 Nov 8;17(S2):332. | Qualitative results only |
| 39 | Holden MG, Pierce A, Pham A, et al. Fertility preservation counselling and referral rates prior to initiation of gender affirming hormone therapy in transgender and non-binary adolescents. *Fertility and Sterility* 2023;120(4):Supp E160-1 | Conference abstract |
| 40 | Ingraham N, Hann L. ‘Stigma R us’: Stigma management at the intersection of abortion care and transgender care in family planning clinics. *SSM - Qualitative Research in Health*, 2022;2:100043 | Healthcare workers only |
| 41 | Ivanenko P, Kim Y, Weinstein M. Perioperative outcomes following combined gender-affirming vaginectomy and hysterectomy compared to vaginectomy only. *International Urogynecology Journal* 2022;28(6S):S1-S285 | No pregnancy data |
| 42 | James-Abra S, Tarasoff LA, Green D, et al. Trans people’s experiences with assisted reproduction services: a qualitative study. *Human Reproduction* 2015;30(6):1365–1374, | Not specific to trans men |
| 43 | Johansson M, Wirén A, Ssempasa D, et al. A qualitative study of midwives’ challenges to support transmen during childbirth: A short report. *European Journal of Midwifery.* 2020;7;4:3 | Healthcare workers only |
| 44 | John JN, Martin ZC. Abortion needs expressed on Reddit after the Dobbs v. Jackson Women’s Health Organization decision in the United States. *Perspectives in Sexual and Reproductive Health* 2024; 56(1):41-49 | Not specific to trans men |
| 45 | Johnson A. Barriers influencing decision to pursue fertility preservation prior to gender affirming surgery among transgender men/gender non-binary persons. *Fertility and Sterility* 2023;120(4):Supp E158-9 | Does not involve pregnant trans men |
| 46 | Johnson A, McClurg AB, Baldino JN, et al. Decisional regret regarding preoperative fertility preservation choices after gender-affirming surgery in transgender men/gender nonbinary persons. *Obstetrics & Gynecology* 2025;145(6S):12S | Does not involve pregnant trans men |
| 47 | Johnson A, Baldino J, Peavey MC, et al. Live birth following IVF pregnancy in a transgender man of advanced paternal age with a history of prolonged testosterone use. *Fertility and Sterility* 2022;118(4)Supp e233 | Case report |
| 48 | Jones RK, Witwer E, Jerman J. Transgender abortion patients and the provision of transgender-specific care at non-hospital facilities that provide abortions. *Contraception X*. 2020;2:100019. | Not an actual number of pregnancies, but an estimate of number of abortions |
| 49 | Kanj R, Conard L, Corathers S, et al. Hormonal contraceptive choices in a clinic-based series of transgender adolescents and young adults. *The International Journal of Transgenderism* 2019;*20*(4):413–420. [https://doi.org/10.1080/15532739.2019.1631929](https://protect.checkpoint.com/v2/r02/___https://doi.org/10.1080/15532739.2019.1631929___.YzJlOmFuZ2xpYXJ1c2tpbnVuaXZlcnNpdHk6YzpvOmNmYWYxNDhkYTExYzQ2N2NkYmJjYTNmZjdjNTY5ZWU2Ojc6MjhkYzpmODlhZjliODY4NzhiOTNjMTczZjlhODZhNGQ3OTNmMWEyYjllYWJiZWI3OWFmMWExOTkyMTU0NjBkYTZkYzY3OnA6VDpG) | Risk but not experience of pregnancy reported |
| 50 | Kasten Z, Lujan S, Jakeman B, et al. Contraceptive use in patients with gender dysphoria who were assigned female at birth receiving care at a specialty gender-affirming clinic. *Journal of the American Pharmaceutical Association* 2024;64(1):273-277. | Not specific to pregnant trans men |
| 51 | Kawsar M, Linander I. "It's a patient safety issue" A qualitative study with care professionals on their experiences of meeting trans people in obstetric and gynaecological care. *Sexual and Reproductive Healthcare* 2022;34:100786. | Not specific to pregnant trans men |
| 52 | Kett PM, van Eijk MS, Guenther GA, et al. "This work that we're doing is bigger than ourselves": A qualitative study with community-based birth doulas in the United States. *Perspectives in Sexual & Reproductive Health*. 2022;54(3):99-108. | No data on pregnant trans men |
| 53 | Kirubarajan A, Barker LC, Leung S, et al. LGBTQ2S+ childbearing individuals and perinatal mental health: A systematic review. *BJOG*. 2022;129(10):1630-1643. | Review |
| 54 | Klittmark S, Malmquist A, Karlsson G, et al. When complications arise during birth: LBTQ people's experiences of care. *Midwifery*. 2023;121:103649 | Does not involve transmasculine pregnancy |
| 55 | Kreines F, Cox C, Harvie H, et al. Lower urinary tract symptoms in transmasculine individuals: prevalence and association with anxiety and depression. *American Journal of Obstetrics and Gynecology* 2024;230(4):S1199-S2000 | Conference abstract |
| 56 | Kyweluk MA, Kirkley J, Grimstad F, et al. Desire for genetically related children among transgender and gender-diverse patients seeking gender-affirming hormones. *Fertility and Sterility Reports* 2023;4(2):224-230. | Not specific to trans men’s pregnancies |
| 57 | La V, Raidoo S, Termulo K, et al. Knowledge and Provision of Care to Transgender People by Obstetrician-Gynecologists in Hawai'i. *Hawaii Journal of Health & Social Welfare* 2022;81(10):272-278. | No specific data on transmasculine pregnancies |
| 58 | Lacombe-Duncan A, Andalibi N, Roosevelt L, et al. Minority stress theory applied to conception, pregnancy, and pregnancy loss: A qualitative study examining LGBTQ+ people's experiences. *PLoS One*. 2022;17(7):e0271945. | Not specific to trans men and pregnancy |
| 59 | Lazaris E, Sanders J, Carter G, et al. Contraceptive uptake amongst gender-expansive individuals in the HER Salt Lake Contraceptive Initiative. *Contraception* 2022;116:P90 | Conference abstract |
| 60 | Lipkin P, Monseur B, Mayo JA, et al. Effect of prior training on reproductive endocrinologist and infertility specialists’ knowledge, skills, attitudes and behaviours regarding the care of transgender and gender diverse individuals *Fertility and Sterility* 2022;118(5):Supp E49-50 | Conference abstract |
| 61 | Liu C, Underhill K, Aubey JJ, et al. Disparities in mistreatment during childbirth. J*AMA Network Open*. 2024;7(4):e244873. | Not specific to pregnant trans men |
| 62 | Lothstein LM. Female-to-male transsexuals who have delivered and reared their children. *Annals of Sex Research.* 1988;1(1):151–66. | Before searches started |
| 63 | Lowik AJ, Al-Anzi SM, Amarasekera A et al. Transgender youths perspectives on factors influencing intended and unintended pregnancies. *Women’s Reproductive Health* 2023;10(4):572-90 | No experienced pregnancies |
| 64 | MacDonald TK. Lactation care for transgender and non-binary patients: Empowering clients and avoiding aversives: *Journal of Human Lactation*, 2019;*35*(2), 223–226. [https://doi.org/10.1177/0890334419830989](https://protect.checkpoint.com/v2/r02/___https://doi.org/10.1177/0890334419830989___.YzJlOmFuZ2xpYXJ1c2tpbnVuaXZlcnNpdHk6YzpvOmNmYWYxNDhkYTExYzQ2N2NkYmJjYTNmZjdjNTY5ZWU2Ojc6NjUwZjpmNTA3ZjUzNjY2ZGU4MzQ4MTZlN2VjNTAwZDk3ODFkOGU5ZmI3ZjFlOGJhMWJiMmQ2YWVhZjE1ODhmNjQ5M2YwOnA6VDpG) | Commentary article with no original data. |
| 65 | MacLean LR. Preconception, pregnancy, birthing, and lactation needs of transgender men. *Nursing for Women's Health* 2021; 25(2):129-138 | Editorial |
| 66 | Malmquist A, Jonsson L, Wikström J, et al. Minority stress adds an additional layer to fear of childbirth in lesbian and bisexual women, and transgender people. *Midwifery*, 2019;*79*, 102551. [https://doi.org/10.1016/J.MIDW.2019.102551](https://protect.checkpoint.com/v2/r02/___https://doi.org/10.1016/J.MIDW.2019.102551___.YzJlOmFuZ2xpYXJ1c2tpbnVuaXZlcnNpdHk6YzpvOmNmYWYxNDhkYTExYzQ2N2NkYmJjYTNmZjdjNTY5ZWU2Ojc6YzI1ZTo2NmZjZTlkMTRiYjI1ZjJkYjQ4M2MxMjBiNDdiYWY0NTczZjEzMmVhN2U4Yzg0YmNkNjhiNTVhZjUzOGUwZTU1OnA6VDpG) | Transgender participants had no reports of pregnancy among them |
| 67 | Malmquist A, Nieminen K. Negotiating who gives birth and the influence of fear of childbirth: Lesbians, bisexual women and transgender people in parenting relationships. *Women and Birth*, 2021;*34*(3), e271–e278. | Transgender participants had no reports of pregnancy among them |
| 68 | Malmquist A, Wikström J, Jonsson L, et al. How norms concerning maternity, femininity and cisgender increase stress among lesbians, bisexual women and transgender people with a fear of childbirth. *Midwifery.* 2021;93:102888. | Does not involve pregnant |
| 69 | Mann S. The Labour Force Status of Transgender People and The Impact of Removing Surgical Requirements to Change Gender on ID Documents. GLO Discussion Paper, No. 670. Essen; 2020. | About trans men having children not pregnancy/births |
| 70 | Mascarenhas RNDS, Santos VVC, Santana BS, et al. Trans men and paternal pregnancy: experiences during the pregnancy-puerperal period. Cien Saude Colet. 2024;29(4):e16172023. | Case report |
| 71 | Maxwell S, Noyes N, Keefe D, et al. Pregnancy outcomes after fertility preservation in transgender men. *Obstetrics & Gynecology*, 2017;*129*(6):1031–1034. [https://doi.org/10.1097/AOG.0000000000002036](https://protect.checkpoint.com/v2/r02/___https://doi.org/10.1097/AOG.0000000000002036___.YzJlOmFuZ2xpYXJ1c2tpbnVuaXZlcnNpdHk6YzpvOmNmYWYxNDhkYTExYzQ2N2NkYmJjYTNmZjdjNTY5ZWU2Ojc6ZjhiMzo2ZTU5MzdhZmQyYjk2ZmRkY2I5OTBiNmY1NjJhMWEyNjBjMmRkMTcwOTg3ZWNhYTM3NTVjNDcxMDAxZWQzNmIwOnA6VDpG) | Transgender participants were not gestational parents |
| 72 | McCann E, Brown B, Hollins-Martin C, Murray K, McCormich F. The views and experiences of LGBTQ+ people regarding midwifery care: A systematic review of the international evidence. Midwifery 2021;103:103102 | Review |
| 73 | McDilda KG, Walker ZW, Lanes A, et al. Thoughts and opinions about fertility preservation and family building from the transgender community- an interview based approach. *Fertility and Sterility* 2023;120(4): Supp E156 | Conference abstract |
| 74 | McMillian-Bohler J, Gedzyk-Neiman S, Hepler B, et al. The power of a story: Enhancing students' empathy for transgender pregnant men. *Journal of Nursing Education.* 2022;61(8):489-492. | Healthcare workers’ education only |
| 75 | Mehra G, Boskey ER, Peters CJ, et al. Assessing fertility intentions in patients presenting for gender-affirming surgery. *LGBT Health* 2022;9(5):325-332 | No data on pregnancies |
| 76 | Moayedi G, Lee S, Soon R, et al. Unmet reproductive health needs of transgender and gender diverse people in Hawai'i: A qualitative needs assessment. *Hawaii Journal of Health & Social Welfare.* 2024;83(3):68-74 | No pregnant transmen used in the study |
| 77 | Moravek MB, Kinnear HM, George J, et al. Impact of exogenous testosterone on reproduction in transgender men. *Endocrinology* 2020;161:1–13 | Review |
| 78 | Morrison A, Olezeski C, Cron J, et al. A pilot study to assess attitudes toward future fertility and parenthood in transgender and gender expansive adolescents. *Transgender Health* 2020;*5*(2),129–137. [https://doi.org/10.1089/TRGH.2019.0075](https://protect.checkpoint.com/v2/r02/___https://doi.org/10.1089/TRGH.2019.0075___.YzJlOmFuZ2xpYXJ1c2tpbnVuaXZlcnNpdHk6YzpvOmNmYWYxNDhkYTExYzQ2N2NkYmJjYTNmZjdjNTY5ZWU2Ojc6NjZmMDplY2IyNzY3YThjM2JmYTk3MTBjZTMxYjllNmM3MDgyODA0NTRiMjk0MTlkNGM2ZGNlNDE0NGRkMThjMzM3NjE5OnA6VDpG) | No pregnancy-related data |
| 79 | Moxley R. Affirming pregnancy care for transgender and gender-diverse patients. *Canadian Family Physician* 2023;69(6):407-408 | No data on pregnant trans men |
| 80 | Murdock M. Providing inclusive midwifery care for 2SLGBTQQIA+ people: Supporting inclusion in Ontario's midwifery education program. Journal of Midwifery & Women’s Health. 2024;69(1):91-100 | Not specific to trans men |
| 81 | Nahata L, Chen D, Quinn GP, et al. Reproductive attitudes and behaviors among transgender/nonbinary adolescents. *Journal of Adolescent Health* 2020;*66*(3), 372–374. [https://doi.org/10.1016/J.JADOHEALTH.2019.09.008](https://protect.checkpoint.com/v2/r02/___https://doi.org/10.1016/J.JADOHEALTH.2019.09.008___.YzJlOmFuZ2xpYXJ1c2tpbnVuaXZlcnNpdHk6YzpvOmNmYWYxNDhkYTExYzQ2N2NkYmJjYTNmZjdjNTY5ZWU2Ojc6Mzk5NjowNmMxNjI0ZThlMGIzM2MzOTJhY2IzNmM0MzNhOTQ0MjY5ODEzMTBlYzZiMzI4ODEyOTRhYjA2OGE1NjZkZDY1OnA6VDpG) | No reports of pregnancy among transgender participants |
| 82 | Norton J. Transmasculine chestfeeding: 1. Infant feeding and transgender men: considering our own perceptions. *The Practising Midwife* 2022;25(2): https://doi.org/10.55975/CJVW6623 | Editorial |
| 83 | Norton J. Transmasculine chestfeeding: 2. Chestfeeding and mental health: what do we understand? *The Practising Midwife* 2022;25(3):https://doi.org/10.55975/ZUSX6380 | Editorial |
| 84 | Obedin-Maliver J, Makadon HJ. Transgender men and pregnancy. *Obstetric Medicine* 2016;9(1):4–8 | Review |
| 85 | O’Brien KE, Farahzad MM, Rosen MW, et al. Oophorectomy at the time of gender affirming hysterectomy in young adults. J*ournal of Pediatric and Adolescent Gynecology* 2023;36(2):221 | Conference abstract |
| 86 | O’Docherty A, Wreglesworth L, Volikas I. Putting the ‘T’ in Inclusivity- improving perinatal care for transgender people. *International Journal of Obstetric Anesthesia* 2022;P91 | Conference abstract |
| 87 | Parikh N, Gargollo P, Granberg C. Health Care Disparities in the Transgender Population. *The Journal of Sexual Medicine* 2023;20(Supp1):qdad060.258 | Conference abstract |
| 88 | Parker G, Kelly L, Miller S. Taking up the challenge of trans and non-binary inclusion in midwifery education: Reflections from educators in Aotearoa and Ontario, Canada. *Midwifery* 2023:118:103605 | About education of health professionals |
| 89 | Parker G, Kelly L, Miller S, et al. Perinatal care providers' preparedness for working with transgender and non-binary clients in Aotearoa New Zealand: survey findings from the trans pregnancy care project. *Journal of Paediatrics and Child Health* 2023;118;103605 | Healthcare workers only |
| 90 | Permezel J, Arnold AS, Thomas J, et al. Experiences in the delivery of preconception and pregnancy care for LGBTIQA+ people: A systematic review and thematic synthesis of patient and healthcare provider perspectives. *Midwifery* 2023;123:103712 | Review |
| 91 | Pickering S, Manze M, Losch J, et al. Delays in obtaining abortion and miscarriage care among pregnant persons in New York State during the COVID-19 pandemic: The CAP Study. *Women’s Health Reports* 2024;5(1):30-39. | Not specific to trans men |
| 92 | Pyne J, Bauer G, Bradley K. Transphobia and other stressors impacting trans parents. *Journal of GLBT Family Studies*, 2015;11(2):107–126. [https://doi.org/10.1080/1550428X.2014.941127](https://protect.checkpoint.com/v2/r02/___https://doi.org/10.1080/1550428X.2014.941127___.YzJlOmFuZ2xpYXJ1c2tpbnVuaXZlcnNpdHk6YzpvOmNmYWYxNDhkYTExYzQ2N2NkYmJjYTNmZjdjNTY5ZWU2Ojc6MjQ5MjphZTM4ZWVlMjcwZDljODMyZWJkYWYwMmVjNzE1MTljOTc1MzgyYzc5YzgzYTRiMjZiZjk1NjhhYjMwYWNjOGVjOnA6VDpG) | Parents not disaggregated by trans men/women |
| 93 | Quinn V, Nash R, Hunkeler E, et al. Cohort profile: study of transition, outcomes and gender (STRONG) to assess health status of transgender people. *BMJ Open*, 2017;*7*(12). [https://doi.org/10.1136/BMJOPEN-2017-018121](https://protect.checkpoint.com/v2/r02/___https://doi.org/10.1136/BMJOPEN-2017-018121___.YzJlOmFuZ2xpYXJ1c2tpbnVuaXZlcnNpdHk6YzpvOmNmYWYxNDhkYTExYzQ2N2NkYmJjYTNmZjdjNTY5ZWU2Ojc6MDZjODpkMzk5YjA0MjZiZGY5YjZhZDUzZjRiZmRlN2M2ZTVkMjA5ZDk5YWRjNGQ3Nzc4YWU3YmY1MmVjN2I2NzFkM2YxOnA6VDpG) | Pregnancy history not reported |
| 94 | Rodriguez-Wallberg K, Obedin-Maliver J, Taylor B, et al. Reproductive health in transgender and gender diverse individuals: A narrative review to guide clinical care and international guidelines. *International Journal of Transgender Health* 2023;24(1):7–25 | Review |
| 95 | Rubin ES, Yang Y, Qiu M, et al. Multicenter case series of transgender men with fertility benefits: Access to care and navigating obstacles. *Fertility and Sterility* 2022;118(4):Supp E22 | Conference abstract |
| 96 | Schnell A. Successful Co-Lactation by a Queer Couple: A Case Study. *Journal of Human Lactation*. 2022;38(4):644-650 | Case report |
| 97 | Silva GC, Puccia MIR, Barros MNDS. Transsexual men and pregnancy: an integrative literature review. *Cien Saude Colet.* 2024;29(4):e19612023. | Review |
| 98 | Singer RB, Crane B, Lemay EP, et al. Improving the knowledge, attitudes, and behavioral intentions of perinatal care providers toward childbearing individuals identifying as LGBTQ: A quasi-experimental study. *Journal of Continuing Education in Nursing*, 2019;*50*(7):303–312. [https://doi.org/10.3928/00220124-20190612-05](https://protect.checkpoint.com/v2/r02/___https://doi.org/10.3928/00220124-20190612-05___.YzJlOmFuZ2xpYXJ1c2tpbnVuaXZlcnNpdHk6YzpvOmNmYWYxNDhkYTExYzQ2N2NkYmJjYTNmZjdjNTY5ZWU2Ojc6NmUzMjo5ZGI4NDBmNjg2M2RhM2M2OTA4OTFhZTJjNzY1Y2Q1MmViNGNiYmViN2ZlNzY0MWU2MmJiMDUwZWMyOGIwNjhjOnA6VDpG) | Healthcare workers’ education only |
| 99 | Smith AL, Chen J, Wyman JF, et al. Survey of lower urinary tract symptoms in United States women using the new lower urinary tract dysfunction research Network-Symptom Index 29 (LURN-SI-29) and a national research registry. *Neurourology & Urodynamics.* 2022;41(2):650-661. | Does not include pregnant trans men |
| 100 | Spizzirri G, Eufrásio R, Lima MC, et al.Proportion of people identified as transgender and non-binary gender in Brazil. *Scientific Reports*, 2021;11(1), 1–7. [https://doi.org/10.1038/s41598-021-81411-4](https://protect.checkpoint.com/v2/r02/___https://doi.org/10.1038/s41598-021-81411-4___.YzJlOmFuZ2xpYXJ1c2tpbnVuaXZlcnNpdHk6YzpvOmNmYWYxNDhkYTExYzQ2N2NkYmJjYTNmZjdjNTY5ZWU2Ojc6NWM1ZDozNGUwN2YyYWI0ZDNhYjhhNjEzMDY4ZTJkMjhjZGY1MjFmOWJkNDM2YTlmYzM2MWJmOTBkN2E0MDViMmQxNGZkOnA6VDpG) | Analyses not disaggregated by sex. |
| 101 | Steininger J, Knaus S, Kaufmann U, et al. Treatment trajectories of gender incongruent Austrian youth seeking gender-affirming hormone therapy. *Frontiers in Endocrinology* 2024;15:1258495. | Does not include pregnant trans men |
| 102 | Stotzer RL, Herman JL, Hasenbush A. *Transgender Parenting: A Review of Existing Research*. 2014 <https://williamsinstitute.law.ucla.edu/wp-content/uploads/Trans-Parenting-Review-Oct-2014.pdf> | Review |
| 103 | Stroumsa D, Crissman H, Moniz MH. Pregnancy outcomes in transgender people-reply. *JAMA.* 2023;330(16):1588. | Not original research -comment to editor |
| 104 | Sundaram V, Stark B, Jaswa E, et al. Decision regret, and other mental health outcomes, following fertility preservation in the transgender individual compared to the cisgender woman. *Journal of Assisted Reproduction and Genetics* 2024; 41(4):1077-1085 | Does not involve pregnant transmasculine people |
| 105 | Tarasoff L, James-Abra S, Ross L, et al. Trans people’s experiences with assisted reproduction services: A qualitative study. *Human Reproduction*, 2015;30(6),1365–1374. | Transgender participants were not yet pregnant. |
| 106 | Thibault MD, Southworth E, Fairchild P, et al. Perioperative outcomes associated with gender-affirming vaginectomy: A cohort study from a single institution. *American Journal of Obstetrics & Gynecology* 2021;224(6):S744-S745 | Conference abstract |
| 107 | Thomas EG, Goodarzi B, Frese H, et al. Pregnancy experiences of transgender and gender expansive individuals: A systematic scoping review from a critical midwifery perspective. *Birth.* 2024;00:1–14. | Review |
| 108 | Tishelman AC, Sutter ME, Chen D, et al. Health care provider perceptions of fertility preservation barriers and challenges with transgender patients and families: qualitative responses to an international survey. *Journal of Assisted Reproduction and Genetics* 2019;*36*(3),579–588. [https://doi.org/10.1007/s10815-018-1395-y](https://protect.checkpoint.com/v2/r02/___https://doi.org/10.1007/s10815-018-1395-y___.YzJlOmFuZ2xpYXJ1c2tpbnVuaXZlcnNpdHk6YzpvOmNmYWYxNDhkYTExYzQ2N2NkYmJjYTNmZjdjNTY5ZWU2Ojc6ZDY1YzplOGE0ZTYyNzM1OTkzZTBkMDhmYmZiYmQzYzkxMWQyZjk0NjRhODZmNTEwYTg1NzhkOTg5NDIyOGU4NTJjZWFmOnA6VDpG) | Fertility paper. No relevant data |
| 109 | Tyrie D, Oliva A, Llorin H, et al. Transgender and gender diverse individuals' perspectives on discussions of fetal sex chromosomes in obstetrics care. Journal of Genetic Counselling. 2024;33:1271–1284 | Qualitative results only |
| 110 | von Doussa H, Power J, Riggs D. Imagining parenthood: the possibilities and experiences of parenthood among transgender people. *Culture, Health & Sexuality* 2015;17(9), 1119–1131. [https://doi.org/10.1080/13691058.2015.1042919](https://protect.checkpoint.com/v2/r02/___https://doi.org/10.1080/13691058.2015.1042919___.YzJlOmFuZ2xpYXJ1c2tpbnVuaXZlcnNpdHk6YzpvOmNmYWYxNDhkYTExYzQ2N2NkYmJjYTNmZjdjNTY5ZWU2Ojc6YjhmNjo1YzNkMGFkMTc3ODcyOTgyNDQ2MjFmMDBmYWI3NmZiYTY2ZTA3N2NhODNkMDIxYjEyOTc5YjE0MzJhNjFjYmI3OnA6VDpG) | Trans men were not gestational parents |
| 111 | Voultsos P, Zymvragou CE, Karakasi MV, et al. A qualitative study examining transgender people’s attitudes towards having a child to whom they are genetically related and pursuing fertility treatments in Greece. *BMC Public Health 2021;21*(1):1–17. [https://doi.org/10.1186/S12889-021-10422-7](https://protect.checkpoint.com/v2/r02/___https://doi.org/10.1186/S12889-021-10422-7___.YzJlOmFuZ2xpYXJ1c2tpbnVuaXZlcnNpdHk6YzpvOmNmYWYxNDhkYTExYzQ2N2NkYmJjYTNmZjdjNTY5ZWU2Ojc6OWNmNzo3MTdlYzRmZjgzMmJlYWNkNjE1N2JkMzVkNDk3Njc1YzNjZmY3ODRmNmM2MDQ1MzlhYzJmZTBjMzVhMTI2MDUyOnA6VDpG) | Case report |
| 112 | Webb K, Rickford R, Edun C, et al. Trans and non-binary experiences of maternity services: cautioning against acting without evidence. *British Journal of Midwifery*, 2023;31(9):512-8 | Commentary article with no original data presented |
| 113 | White J, Jackson A, Druce I, et al. Oocyte cryopreservation and reciprocal in vitro fertilization in a transgender man on long term testosterone gender-affirming hormone therapy: a case report. Fertility and Sterility Reports 2023;10;5(1):111-113 | Case report |
| 114 | Wiesemann C, Frentz HM. Schwangerschaft transgeschlechtlicher Personen: eine Herausforderung für die Frauenheilkunde und Geburtshilfe. *Geburtshilfe Und Frauenheilkunde*, 2020;80(7):670–671. [https://doi.org/10.1055/A-1125-8372](https://protect.checkpoint.com/v2/r02/___https://doi.org/10.1055/A-1125-8372___.YzJlOmFuZ2xpYXJ1c2tpbnVuaXZlcnNpdHk6YzpvOmNmYWYxNDhkYTExYzQ2N2NkYmJjYTNmZjdjNTY5ZWU2Ojc6ZGE4YTo1NjQ2MGViZWNlN2U3ZGZkOWFiMmZlOTg4YTM2ZmI3OTBmZmQ4Mzg4NWFkNWM4MTFkNDRkNmNlYzUxOGY5ZDI1OnA6VDpG) | Commentary article with no original data presented |
| 115 | Wolfe-Roubatis E, Spatz DL. Transgender men and lactation. *MCN: The American Journal of Maternal/Child Nursing*. 2015;40(1):32–8. | Case reports only |
| 116 | Yoshida A, Kaji T, Imaizumi J, et al. Transgender man receiving testosterone treatment became pregnant and delivered a girl: A case report. The Journal of Obstetrics & Gynecology Research 2022;48(3):866-8 | Case report |

Reports excluded

| **No** | **Reference** | **Reason** |
| --- | --- | --- |
| 1 | Anon. Trans + non binary experiences of maternity services. LGBT Foundation, Manchester 2023. | Unpublished report |
| 2 | Bachmann, C. and Gooch, B. (2018a). LGBT in Britain Trans Report. Stonewall and YouGov. Available at: [https://www.stonewall.org.uk/system/files/lgbt_in_ britain_-_trans_report_final.pdf](https://protect.checkpoint.com/v2/r02/___https://www.stonewall.org.uk/system/files/lgbt_in_%20britain_-_trans_report_final.pdf___.YzJlOmFuZ2xpYXJ1c2tpbnVuaXZlcnNpdHk6YzpvOjMzNTZkNDAxMmZlNDczYTRkY2Q1NWU4YjcwMGZlNzBiOjc6ZGNhYjpkNmZkNTBlYWE1MDQyNWEwZTg1YzZiMTI4ZGNmMDRlZTYzZjA4NzVjZWZlMmI2MzZiMDhlZGQ1OTU3Yzc1ZjE3OnA6VDpG) | Not about pregnancy |
| 3 | Browne K, Scott E-J, Valentine V, et al. (2015). Trans Community Research Final Project Report. University of Brighton & Brighton & Hove LGBT Switchboard. Available at: [http://www.bhconnected.org.uk/content/needs-assessments](https://protect.checkpoint.com/v2/r02/___http://www.bhconnected.org.uk/content/needs-assessments___.YzJlOmFuZ2xpYXJ1c2tpbnVuaXZlcnNpdHk6YzpvOjMzNTZkNDAxMmZlNDczYTRkY2Q1NWU4YjcwMGZlNzBiOjc6MGE2Yjo5NmQ1NDFjMmU2ODY0ZTkyN2Q2OTM2Y2RjNzVhYzJlZDM2NThkZmU0MGIxYzA5YTg3ODJiZTRkNGUzMmMxNmUxOnA6VDpG) | Not about pregnancy |
| 4 | Coe O. Experiences of pregnancy and birthing for trans and non-binary people in the UK. Doctor in Clinical Psychology (DClinPsy) Thesis, Royal Holloway, University of London 2023. | Unpublished report |
| 5 | Davda P. Trans community health profile 2022. Birmingham 2022 | Not about pregnancy |
| 6 | Government Equalities Office. (2018a) National LGBT Survey. Available at: https:// [www.gov.uk/government/publications/national-lgbt-survey-summary-report](https://protect.checkpoint.com/v2/r02/___http://www.gov.uk/government/publications/national-lgbt-survey-summary-report___.YzJlOmFuZ2xpYXJ1c2tpbnVuaXZlcnNpdHk6YzpvOjMzNTZkNDAxMmZlNDczYTRkY2Q1NWU4YjcwMGZlNzBiOjc6MDA0MTo0MjIyMDllMzg3MWE3YmQ5ZDcwNmFlY2RiZGNjMjI4MWFkOTFiZmI3MDE1ZTZlZDc4ODYyODk2YTFiY2E3MTliOnA6VDpG) | Not about pregnancy |
| 7 | Hord L, Medcalf K. Trans people’s experience of healthcare in England. Transforming Futures Partnership. Undated. | Unpublished report |
| 8 | James SE, Herman JL, Rankin S, et al. The Report of the 2015 U.S. Transgender Survey. 2016 [https://transequality.org/sites/default/files/docs/usts/USTS-Full-Report-Dec17.pdf](https://protect.checkpoint.com/v2/r02/___https://transequality.org/sites/default/files/docs/usts/USTS-Full-Report-Dec17.pdf___.YzJlOmFuZ2xpYXJ1c2tpbnVuaXZlcnNpdHk6YzpvOjFlOGYzOGVjNDYzZjBiODQwNDgyZTBmNjU2NGU0NGQ4Ojc6NTdkMTo1NGNmYmY0NzFhYjJkYzg2YTNmMjU2ZWYzOWE4MWY1ODVhZDU4NWE1MDRmMWQxMWIwOWZmMGI1ZWMxMmZjMmFhOnA6VDpG) | No pregnancy-related data |
| 9 | Manchester City Council (2016). Research Study into the Trans Population of Manchester. Undertaken by LGBT Foundation. Available at: https://www.manchester.gov.uk/downloads/download/6603/research_study_into_the_ trans_population_of_manchester | Not about pregnancy |
| 10 | McNeil, J., Bailey, L., Ellis, S., et al. (2012). Trans Mental Health Study 2012. Scottish Transgender Alliance. September 2012. Available at: [https://www.scottishtrans.org/wp-content/uploads/2013/03/trans_mh_ study.pdf](https://protect.checkpoint.com/v2/r02/___https://www.scottishtrans.org/wp-content/uploads/2013/03/trans_mh_%20study.pdf___.YzJlOmFuZ2xpYXJ1c2tpbnVuaXZlcnNpdHk6YzpvOjMzNTZkNDAxMmZlNDczYTRkY2Q1NWU4YjcwMGZlNzBiOjc6NGYyYzo2YTNlZGQ4ZmE4ZDY5NDBlOGJmOThlOTcxNTJmNmFlOTgyNzAxNzI2ODMzNzFlNzA2YTM2YmY2MTc5MGE2MTlmOnA6VDpG) | Not about pregnancy |
| 11 | Rastogi A, Menard L, Miller GH, et al. Health and wellbeing: A report of the 2022 U.S. Transgender Survey. Advocates for Transgender Equality. | Unpublished report |
| 12 | TransActual (2021). Trans Lives Survey 2021: Enduring the UK’s hostile environment. Available at: https://static1.squarespace.com/ static/5e8a0a6bb02c73725b24dc9d/t/6152eac81e0b0109491 dc518/1632824024793/Trans+Lives+Survey+2021.pdf | Not about pregnancy |
| 13 | Vincent B, Petch M, Holti R. Integrating Care for Trans Adults: Review of the Integration of Trans Health. Open University/LGBT Foundation. Undated. | Not about pregnancy |

**Table S2. Characteristics of included studies**

| **Authors**  **(year)**  **Country** | **Methods - data collection/ analysis** | **Participants** | **Setting/ recruitment** | **Sample descriptions** | **Trans and non-trans comparators (when available)** | **Outcomes** | **Study type category** | **Funders** |
| --- | --- | --- | --- | --- | --- | --- | --- | --- |
| Albar et al. (2023)  Saudi Arabia, Canada | Retrospective cohort study | 18 transgender men | Consecutive patients at one IVF clinic  Oct 2019-Apr 2021 | Seeking fertility preservation & on androgens for >1 month | n/a | Quant | C | None reported |
| Baker et al. (2019) USA | Retrospective case note analysis | 340 trans men & masculine centred GNC people | Private surgical practice/ convenience 2013-2018. | Undergoing gender-affirming chest-contouring surgeries | n/a | Quant | C | KFF |
| Brewer & Thomas (2019) USA | Cross-sectional survey random selection | 237 transgender people (sex at birth not specified) | University students responding to 2011–2014 National College Health  Assessment | Answering questions on inter-personal violence | 57,176 females & 27,321 males | Quant | A | Unfunded |
| Cao et al.  (2021)  USA | Retrospective review of surgery notes | 72 transmasculine patients | Undergoing gynaecological procedures at Thomas Jefferson University Hospital between Oct 2016-May 2020 | Undergoing hysterectomy and bilateral salpingo-oophorectomy | n/a | Quant | C | None reported |
| Charlton et al.  (2021)  USA | Semi-structured interviews/ TA immersion and crystallisation | 10 transmasculine people | SLOPE study / multiple sources: support networks, clinics, community centres & events, & online through social media, targeted advertisements & community partners  Mar 2017-Aug 2018 | Had teenage pregnancies or who became unintentionally pregnant later | n/a | Qual | D | National Institutes of Health  NIH (F32HD 084000, F32HD1 00081, R00HD 082340); ACS (MRSG CPHPS 130006); MCHB (6T71MC00009); SFP (SHPRF9-18); AEFPTAHI; & BF. |
| Cipres et al.  (2017)  USA | Retrospective case note analysis | 26 transgender men younger than 50 yrs | Sexual Health Clinic for sex workers/ convenience  2012-2015 | Reported current or past sex work | n/a | Quant | C | UCSF PROF-PATH, NIMHHD |
| Copeland et al.  (2023)  Australia | Case study reports | 2 transgender and non-binary AFAB | Midwifery Group Practice program (no dates) | Received pregnancy and birth care | n/a | Qual | D | No funding |
| Dantas et al.  (2024)  Brazil | Semi-structured interviews | 5 transmasculine people | National health system trans outpatient referral clinic in Manaus, Aug 2022-Apr 2023 | Pregnant before gender transition and whose gender transition was monitored at clinic | n/a | Qual | D | FAPEAM - Brasil |
| Ellis et al.  (2015)  USA | In-depth interviews/  Grounded theory analysis | 8 people (6 reported multiple gender identities e.g., gender variant, two-spirit; 2 identified solely as male) | Health care & social service providers/ convenience & respondent-driven sampling  Sept 2011-May 2012 | Pregnancy within the previous 5 years, not resulting in loss | n/a | Qual | D | NR |
| Falck et al.  (2021) AND Falck et al. (2024)  Sweden | Face-to-face interviews  Inductive thematic analysis of content | 12 transmasculine patients (2021)  12 people (AFAB with a self-identification on the male side of the gender spectrum or as NB before pregnancy.  Some used more than one concept to explain their gender identity (2024). | Swedish Youth Federation for LGBTQ rights, gender experts, Gender clinics & participants from former studies recruited individuals to the study  Dec 2016–Jan 2019 | Prenatal care patients | n/a | Qual and Quant | D | Swedish Research Council  (523-2011-3807) |
| Fischer et al.  (2021)  Canada | Unstructured, tape-recorded interviews | 5 non-binary (AFAB) patients (identifying other than cisgender) | Responded to recruitment on social media platforms,  (no dates) | All birthed a child | n/a | Qual | D | None reported |
| Fix et al.  (2020)  USA | Semi-structured interviews /  Thematic analysis | 5 TGE-identified people (subgroup of sample) | Potential stakeholders that authors had identified approached via email & SM/ snowball using SM & interviewees’ contacts  Oct 2017-Jan 2018 | Had accessed contraception or abortion | n/a | Qual | B | SFP (SFPRF11-II1); NIDDKD (K12DK111028) |
| Galvao et al.  (2024)  Brazil | Interviews | 2 trans men | Human milk bank  Aug-Oct 2022 | Users of chest feeding service | n/a | Qual | D | None reported |
| Ghofranian et al. (2022) AND  Ghofranian et al. (2023)  USA | Data collection from medical records | 32 trans men patients, 77 trans men patients and partners underwent fertility counselling | Single academic infertility centre  Jan 2013-Dec 2021 | History of gender-affirming hormone therapy undergoing fertility treatments | n/a | Quant | B | No funding |
| Gomez et al.  (2020)  USA | Semi-structured interviews/  Thematic analysis | 20 people AFAB (13 transgender men, 4 NB or GQ, 4 identified as men, & 2 as another gender identity) | SM & by using flyers in social service agencies serving LGBTQ people/ convenience sample  (no dates) | Sought reproductive health services in the previous year, or experienced a pregnancy or had sex with a cisgender man in the previous three years | n/a | Qual | B | NR |
| Grimstad et al. (2023)  USA | Retrospective cohort study using chart review | 50 transmasculine patients | All patients 2016-2021 at Cleveland Clinic and 2019-2021 at Boston Children’s Hospital | Had received testosterone and undergone a hysterectomy for gender affirmation purposes | n/a | Quant | C | NR |
| Grimstad et al. (2019)  USA | Retrospective chart review/case note analysis | 94 transmasculine people | All patients of 5 surgical centres treated between 2015-2017 | Had received testosterone and subsequently underwent hysterectomy for gender affirmation purposes | n/a | Quant | B | NR |
| Hawkins et al.  (2021)  USA | Retrospective case series using a billing database | 81 gender affirmation patients | One private and one public safety net hospital.  2000-2018 | Preoperative testosterone use and undergone hysterectomy for gender affirmation | n/a | Quant | C | Supported by US Department of Veteran Affairs Office of Academic Affiliations Fellowship in  Women’s Health |
| Jeftovic et al.  (2018)  Serbia | Case series  (data collection not described) | 124 female to male transsexuals | Not described, presume surgical hospital(s)  Jan 2012-Mar 2017 | Undergoing hysterectomy with bilateral salpingo-oophorectomy, followed by colpocleisis and gender affirmation surgery | n/a | Quant | B | MSTDRS (175048) |
| Leonard et al.  (2022)  USA | Population-based cohort study | 498 birthing fathers with any partner | Linked birth certificate to hospital discharge data 2016-2019 | Live births (excluded multifetal births) | a. mother-father partnerships (1,483,119)  b. mother-mother partnerships (2,572) | Quant | D | Stanford Maternal and Child Health Research Institute |
| Leung et al. (2019)  USA | Retrospective case-control, matched with five unique cisgender patients with either male-factor or tubal-factor infertility. Used database search of electronic records with notes analysis | 26 FtM transgender men | IVF clinic sample  Jan 2010-Jul 2018 | Completed an ovarian stimulation cycle for oocyte cryopreservation, embryo cryopreservation, or intended uterine transfer | 130 females | Quant | B | NR |
| Light et al. (2018)  USA & online | Cross-sectional survey | 196 people self-identified along the masculine spectrum (transgender, transman, TM, ‘FtM’) | 6 LGBT health centres, 26 transgender healthcare providers and 14 online listservs and Facebook groups for transgender men  convenience  Jul-Oct 2016 | Willing to answer survey on general health, use of masculinizing hormones (testosterone)  and contraceptives, fertility, and pregnancy history | n/a | Quant | B | MWHCOGME |
| Light et al. (2014)  USA & online | Cross-sectional survey  Qual survey responses used Grounded theory analysis | 41 transgender men (AFAB with a masculine, TM, transmale, or ‘FtM’ gender identity) | Online survey/ convenience sample with snowball & then respondent driven.  Mar-Dec 2013 | Pregnancy after transition | n/a | Qual/ quant | D | NR |
| MacDonald et al. (2021) AND MacDonald et al. (2016)  Canada | Semi-structured interviews Interpretive description analysis | 22 transmasculine people | Self-selecting convenience sample via social media.  Sept 2014-Oct 2015 | Had experienced or were experiencing pregnancy, birth, and infant feeding | n/a | Qual | D | Canadian Institutes of Health Research |
| Maheux et al. (2021)  USA | Cross-sectional survey | 1,223 gender minority people aged 14-18 (654 trans boys, 399 NB AFAB) | Larger study of adolescent mental health via SM / purposive trans sub-sample  Jul–Oct 2018 | Self-identified gender minority boys or non-binary AFAB | 68 trans girls, 48 non-binary AMAB and 54 questioning/ unsure | Quant | E | UPCRDF; NIMH (T32 MH018951, K01 MH117142 & T32 MH018269); NSFGRF (1940700) |
| Malmquist et al. (2021)  Sweden | Semi-structured interviews/  Inductive thematic analysis | 2 transgender men | SM groups for LGBT families/ convenience (data collected in 2010) | LGBT+ group enrolled because of fear of conception | 15 lesbians, bisexual and non-binary women | Qual | B | RSAS (AM2017-0005) |
| Mattelin et al.  (2022)  Sweden | Retrospective cohort study | 164 transgender men of fertile age | Tertiary centre, case notes using (ICD)-10 codes for gender dysphoria (F64.0, F64.8, 64.9)  2013-2018 | Consultations concerning fertility preservations | 78 transgender women of fertile age | Quant | B | Grant from Swedish state, Hjalmar Svensson’s Research Foundation |
| Moseson et al. 2020, AND  Moseson et al. 2021 AND Moseson et al. 2022  USA | Online cross sectional surveys | 1964 Transgender, non-binary or gender expansive participants, aged 18-45 (PRIDE), 18+ (general population survey) | PRIDE survey and General population survey, recruited through social media, shared via community email lists, and distributed at in-person community events and SRH conferences  2019 | Having trans, non-binary or gender expansive experience | n/a | Quant and Qual | B | Society of Family Planning, National Institute of Diabetes, Digestive and Kidney Disorders, National Institute on Drug Abuse |
| Obedin-Maliver et al. (2017)  USA | Retrospective cohort study | 33 transgender men | Academic hospital in SF billing data & hand review of medical records/ convenience  2000-2012 | Having a hysterectomy for benign indication | 850 cisgender women | Quant | C | UCSFCTSI |
| O’Hanlan et al. (2007)  USA | Retrospective case control study | 41 female-to-male transexuals | Private surgical practice/ convenience analysis using case notes  1996 onwards | Having a hysterectomy, salpingo-oophorectomy and appendectomy | 552 female patients | Quant | C | NR |
| Pfeffer et al.  (2023) AND Riggs et al. (2021) AND Riggs et al. (2020)  Australia, Canada, EU, UK, USA | Semi-structured interview and focus group  Thematic analysis | 70 trans/masculine & NB people (Pfeffer 2023)  Subset of 51 (Riggs 2021)  Subset of 16 (Riggs 2020) | Social media community networks/ convenience  Jun 2018–Oct 2019 | 70 having conceived or willing to discuss conception after coming out as trans  51 who were gestational parents  16 who had experienced a pregnancy loss | n/a | Qual | D | Economic and Social Research Council (UK) |
| Reynolds et al.  (2021)  USA | Questionnaire | 800 transmasculine and non-binary college students | National College Health Assessment Autumn 2015-Spring 2018 | In receipt of pregnancy prevention information and unwanted pregnancies | 185,658 assigned females at birth | Quant | A | American Cancer Society |
| Riggs et al.  (2016) Australia | Cross-sectional survey | 46 trans men & 26 gender diverse people | SM (Facebook)  Jan-Aug 2014 | Interested in completing a survey on parenting | 88 trans women | Quant | B | ARCFF (FT130100087) |
| Riggs & Bartholomaeus (2018)  Australia | Scoping mixed-methods online study | 131 trans men & 149 NB & 32 agender adults | Trans & NB SM groups/ convenience  Jan–Feb 2018 | Decision-making about fertility preservation | 97 trans women | Quant | B | ARCFF (FT130100087) |
| Stark et al.  (2019)  USA | Cross-sectional survey | 150 people (115 trans men & 35 NB) | LGBT+ community health centre, medical record / purposive sample  Mar 2015-Sept 2016 | Using the Fenway Health Centre and sexually active in previous 3 years | n/a | Quant | C | PCORI; ACS |
| Stroumsa et al.  (2023)  USA | Retrospective cohort study | 1907 trans men (256 Medicare, 1651 commercial insurance) | Truven MarketScan Medicaid and commercial databases from 2014-2018 | Had delivered babies | 2,721,507 cis women, (1,255,942 Medicare, 1,465,565 commercial insurance) | Quant | D | NIH and AHRQ grants |
| Tordoff et al. (2019)  USA | Cross-sectional survey | 701 transgender men & 450 GNC people | BRFSS survey (CDC)/ probability sampling from 2014, 2015 and 2016 | To estimate the errors introduced when automated assumptions made about sex/gender identity causing missing responses from participants not being asked sex-specific questions according to body parts correctly | 298 391 cisgender women | Quant | A | NR |
| Van Amesfoort et al.  (2023)  Netherlands | Semi-structured interviews | 5 transgender men | Centre of Expertise on Gender Dysphoria at the University Medical Centres,  Sept 2020–Jan 2021 | Known to have been pregnant when attending medical centre | n/a | Qual | D | No funding |
| Veale et al.  (2016)  Canada | Cross-sectional survey | 923 trans youth of which 540 responded to the question asking about pregnancy involvement | Canadian Trans Youth Health Survey - Youth advisory councils, community organisations, health professionals, SM & word of mouth/ convenience  Oct 2013-May 2014 | Respondents who responded to the question about pregnancy involvement | Respondents who didn’t respond to the question about pregnancy involvement | Quant | D | CIH (MOP 119472) |
| Vyas et al.  (2021)  USA | Multiple choice questionnaire | 397 gender-diverse individuals, of which 187 AFAB, with 24 of these completing follow up survey | UCLA Gender Health Program gender clinic/ convenience  Jan 2018-Mar 2019 | Clinic patients clarifying reproductive desires or intentions | 152 AMAB, with 46 respondents AMAB completing follow up survey | Quant | B | None reported |
| Wierckx et al. (2012)  Belgium | Cross-sectional survey | 50 transsexual men undergoing sex reassignment surgery | Ghent University Hospital Case notes/ consecutive sample  1987-2009 | Reproductive wishes after sex reassignment surgery | n/a | Quant | C | Flemish Foundation of Scientific research |
| Wingo et al. (2018)  USA | Interviews,  Thematic analysis | 7 male/trans men, 13 genderqueer/ gender nonconforming (LGBTQ identified AFAB) | SM/ convenience with subsequent snowballing  Dec 2016-Mar 2017 | Priorities and experiences with reproductive health care | n/a | Qual | B | FFSR (FWO-Vlaanderen) |
| Yaish et al.  (2021)  Israel | Prospective cross-sectional survey | 103 trans men (in two studies) | Clinical (Transgender Health Center)/ convenience  Jun 2014-Jul 2018 | On testosterone (evaluating impact of treatment on reproduction) | n/a | Quant | C | Unfunded |
| Yang et al.  (2023)  China | Cross-sectional study, online questionnaire | 231 transmen parents | Online survey on SM  Jan-Feb 2022 | Transgender and gender-diverse parents and chestfeeding | 361 trans women/55 other trans parents | Quant | D | None reported |

**Notes**:

ACS: American Cancer Society; AEFPTAHI: Aerosmith Endowment Fund for Prevention & Treatment of AIDS & HIV Infections; AFAB/FAAB: (female) assigned (female) at birth; AHRQ: Agency for Healthcare Research and Quality; AMAB: assigned male at birth; ARCFF: Australian Research Council Future Fellowship; BF: Boston Foundation; BRFSS: Behavioural Risk Factor Surveillance System; CDC: Centers for Disease Control & Prevention; CIH: Canadian Institutes of Health; cis: cisgender; FAPEAM: Fundação de Amparo à Pesquisa do Estado do Amazonas; FFSR: Flemish Foundation of Scientific Research; FtM: female to male; GNC: gender non-conforming; GQ: genderqueer; ICD: International Classification of Diseases; IVF: in-vitro fertilisation; KFF: Klarman Family Foundation; LGBTQ: lesbian, gay, bisexual, transgender, queer; LGBT+: lesbian, gay, bisexual, transgender plus other sexual & gender minorities; MSTDRS: Ministry of Science & Technical Development, Republic of Serbia; MtF: male to female; n/a: not applicable; NB: nonbinary/non-binary; MCHB: Maternal & Child Health Bureau; NIDDKD: National Institute of Diabetes, Digestive, & Kidney Disorders; NIH: National Institutes of Health; NIMH: National Institute of Mental Health; NIMHHD: National Institute on Minority Health & Health Disparities; NR: not reported; PCORI: Patient-Centered Outcomes Research Institute; PRIDE: Population Research in Identity and Disparities for Equality; RSAS: Royal Swedish Academy of Sciences; SFP: Society of Family Planning; SLOPE: Sexual Orientation, Gender Identity & Pregnancy Experiences; SM: social media; SRH: sexual & reproductive health; T: testosterone; TGE: transgender (trans), non-binary or/& gender expansive people assigned female or intersex at birth; TM: transmasculine; trans: transgender; UCSF: University of California San Francisco; UCSFCTSI: University of California San Francisco Clinical and Translational Sciences Institute; UPCRDF: University of Pittsburgh Central Research Development Fund; USA: United States of America.

**Table S3. Numerical results from included studies**

| **Authors**  **(year)** | **Study outcome types** | **Age of TNB sample (mean [SD])* or median (range) or range)** | **Results:**  **Pregnancy, parity, birth, Caesarean section** | **Results:**  **Outcomes/ other results of full group** | **Subgroup analyses & demographic assessments** | **Comparator results** |
| --- | --- | --- | --- | --- | --- | --- |
| Albar et al.  (2024) | Quantitative | 27.7 [SD 5.2] | 0% (0/18) had previous pregnancy | Not reported | Not reported | n/a |
| Baker et al.  (2019) | Quantitative | Median 25.5 (IQR 22-30) | 10/158 (2.9%) patients parous  53.5% (182/340) did not report | Parous with testosterone 70% (7/10)  Without testosterone 30% (3/10) | 75% (118/158) White non-Hispanic,  8.8% (14/158) Black or African American | n/a |
| Brewer & Thomas (2019) | Quantitative | 20.0 [NR] range 18-24 | Not reported | 5.1% (12/237) trans participants (mixed AFAB and AMAB) reported that pregnancy was an impediment to academic performance | 65.2% White | 0.59% female & 0.42% male students |
| Cao et al.  (2021) | Quantitative | Median 30, (IQR 19-51) | 60% (43/72) nulliparous  1% (1/72) parous  1% (1/72) ectopic pregnancy  3% (2/72) miscarriage or abortion  1% (1/72) parous and miscarriage  33% (24/72) unknown | 18% (13/72) had endometrial atrophy (all had prior testosterone use)  22% (16/72) had ovarian or paratubal cysts (all had prior testosterone use)  3% 2/72 Caesarean delivery | 76% (55/72) White, 17% (12/72) Black, 6% (4/72) Hispanic, 1% (1/72) Asian | n/a |
| Charlton et al.  (2021) | Qualitative | 34.3 [SD 10.3] range 21 to 53 | Out of 10 patients, 30% (3/10) spontaneous abortions, 40% (4/10) induced abortions, 30% (3/10) live births. | Not reported | 90% (9/10) White,  10% (1/10) Black | n/a |
| Cipres et al.  (2017) | Quantitative | 27.5 [NR] range (24–30.5) | 7.7% (2/26) participants had ≥1 prior pregnancy | Not reported | 62% (16/26) White non-Hispanic 15% (4/26) Hispanic/Latino 8% (2/26) Black/African American  12% (3/26) Asian  4% (1/26) Other | n/a |
| Copeland et al.  (2023) | Qualitative | 21 | 100% (2/2) had been pregnant (primip), 50% (1/2) Caesarean section | Not reported | Not reported | n/a |
| Dantas et al.  (2024) | Qualitative | 29.8 (range 26-38) | 100% (5/5) had had children (no information on parity) | Not reported | Not reported | n/a |
| Ellis et al. (2015) | Qualitative | 33 (range 29-41) | 100% (8/8) had been pregnant  62.5% (5/8) one child  37.5% (3/8) 2 children | 25% (2/8) gave birth at home | 100% white | n/a |
| Falck et al. (2021) AND Falck et al. (2024) | Qualitative | 31 (range 21-40) | 42% (5/12) parous, 58% (7/12) nulliparous  25% (3/12) Caesarean section, 75% (9/12) vaginal delivery, | 50% (6/12) conception via intercourse, 25% (3/12) - inseminations in clinics, 8.3% (1/12) home insemination, 16.7% (2 /12) IVF  58.3% (7/12) postpartum depression  16.7% (2/12) postpartum psychosis  8.3% (3/12) suicide ideation | White 60% (12/20)  67% (8/12) had testosterone, 50% (6/12) mastectomy before pregnancy | n/a |
| Fischer et al.  (2021) | Qualitative | 34.8 (range 31-44) | 40% (2/5) had one child  40% (2/5) had 2 children  20% (1/5) had 3 children | Not reported | 80% (4/5) White  20% (1/5) Multiracial | n/a |
| Fix et al. (2020) | Qualitative | Not reported | Parity proportion unclear | 40% (2/5) accessed abortions | Not reported | n/a |
| Galvao et al.  (2024) | Qualitative | 32.5 | Both service users 100% (2/2) had a natural pregnancy and no previous miscarriages | Not reported | 100% (2/2) White | n/a |
| Ghofranian et al. (2022) AND Ghofranian et al. (2023) | Quantitative | 27.8 (5.8) (n=32)  NR (n=46) | 10.9% (5/46) births with fertility treatment | 1 patient IVF, 1 patient co-IVF, both had live births, 3 IUI and all had live births | Not reported |  |
| Gomez et al.  (2020) | Qual | 26.5, (range 22-29) | Not reported | 15% (3/20) participants had accessed an abortion | 60% (12/20) White  1 each Black, Latinx Asian or Pacific Islander  25% (5/20) Multiracial | n/a |
| Grimstad et al.  (2023) | Quantitative | Median 22, IQR 19-26 | 2% (1/50) previous pregnancy | Not reported | Not reported | n/a |
| Grimstad et al. (2019) | Quantitative | 30.0 [SD 8.6], range 18-53 at hysterectomy | 80.9% (76/94) nulliparous | Not reported | 86.5% (45/94)non-Hispanic white  9.6% (5/94) non-Hispanic black  1 each Asian Pacific Islander, American Indian  44.6% (42/94) missing | n/a |
| Hawkins et al.  (2021) | Quantitative | Median 31 [IQR 27, 40] | 7% (6/81) participants were parous. | 17% (1/81) previous Caesarean sections | Not reported | n/a |
| Jeftovic et al.  (2018) | Quantitative | 28.5 [range 18-43] | 1.6% (2/124) patients were uniparous. All others were nulliparous | Not reported | Not reported | n/a |
| Leonard et al.  (2022) | Quantitative | 30.8 (SD 6.8) | All were pregnant  66% (332/498) had had previous births  (0.03% of the birthing population compared to mother-father pairs) 31% (153/498) Caesarean section | 9.1% (45/498) gestational diabetes mellitus,  8.0% (40/498) hypertensive disorders of pregnancy,  3.8% (19/498) postpartum haemorrhage  8.3% (41/498) preterm birth | 31% (154/498) White  48% (238/498) Hispanic  12% (62/498) Asian/Pacific Island  6% (28/498) multirace  3% (16/498) black | 61% (703,088/1483119) women/men partnerships had had previous births  36% (729/2572) women/women partnerships had had previous births  10.1% (147638/1483119) women/men partnerships 10.7% (272/2572) women/women partnerships gestational diabetes mellitus,  9.1% (135405/1483119) women/men partnerships, 16.2% (417/2572) hypertensive disorders of pregnancy, 4.4% (65810/1483119) women/men partnerships, 8.6% (22/2572) women/women partnerships postpartum haemorrhage, 7.6% (112093/1483119) women/men partnerships, 11.8% (301/2572)  women/women partnerships preterm birth |
| Leung et al. (2019) | Quantitative | 28.3 [SD 6.7] | 3.8% (1/26) had already had a child. | 7.6% (2/26) used IVF – all had live births. | Not reported | 100% (5/5) partners of trans men had live births. Control group results not reported |
| Light et al. (2018) | Quantitative | 31.0 [SD 8.0] | 17% (32/196) participants had been pregnant (total of 60 pregnancies) | Gravidity range 1-8 (but only one was >4 pregnancies).  Most common pregnancy outcomes; delivery > miscarriage > abortion  7 abortions reported (5 who had used testosterone).  18.7% (6/32) had bleeding during pregnancy | Those who had taken testosterone were 3x likelier to have been pregnant (36% vs 13.8%).  88.2% White (173/196)  6.1% Hispanic (12/196)  5.1% Multiracial (10/196)  9.7% Other (19/196) | n/a |
| Light et al. (2014) | Quantitative and qualitative | 28.0 [SD 6.8] | 100% (41/41) were parous  2.4% (1/41) twin birth  30% (12/41) Caesarean section  Mean birthweight 3,146g (SD1,671)  Gestational age at delivery 38 weeks (SD 6 days)  78.0% (32/41) born in hospital, 17.1% (7/41) born at home, 4.9% (2/41) born in independent birth centre  4.9% (2/41) had no prenatal care | 75% (9/12) Caesarean section in those who had used testosterone.  Perinatal complications included  12% (5/41) hypertension,  10% (4/41) preterm labour,  10% (4/41) placental abruption,  7% (3/41) anaemia.  5% (2/41) gestational diabetes | 92% White, 1 each of Asian, Black and Pacific Islander. Those who had used testosterone were significantly less likely to chest feed (40% vs 69%) & CS was more common (36% vs 19%) | n/a |
| MacDonald et al. (2021) AND MacDonald et al. (2016) | Qualitative | Not reported | All had been pregnant,  90% (20/22) carried babies to term,  (5%) 1/22 currently pregnant,  64% (14/22) had one child  (5%)1/22 recent miscarriage  36% (8/22) had previous miscarriages  9% (2/22) had previous abortion | Births occurred between 36 and 42 weeks gestation and birth weights ranged from 2722 to 4308g  Seven participants reported postpartum depression, of whom three received a medical diagnosis.  9 had taken testosterone before they conceived, 8 had started taking it for the first time after their children were born, 5 had never taken it.  41% (9/22) took testosterone before they conceived, 36% (8/22) took after they conceived, 23% (5/22) had never taken testosterone | 59.1% (13/22) White,  9.1% (2/22) Black,  4.5% (1/22) non-Hispanic,  13.6% (3/22) Jewish | n/a |
| Maheux et al. (2021) | Quantitative | 16.0 [range 14-18] | 2.8% (18/654) sexually active trans boys, 4.5% (18/399) sexually active non-binary AFAB adolescents reported having experienced pregnancy (had been pregnant). | Not reported | Proportion White/Caucasian varied between 58.33% and 72.06% across the 5 groups  Also had Black/African American, Hispanic/ Latinx Asian/Pacific Islander and Other or mixed race in each group | 2.44% (1/41 trans girls 3.57 (1/28) non-binary AMAB  had experienced a pregnancy (made someone pregnant)  9.09% (1/22) questioning/unsure had experienced a pregnancy experience |
| Malmquist et al. (2021) | Qualitative | range 25 to 42 | 0% (0/2) births | Not reported | Not reported | 60% (9/15) had births, 3 were pregnant at interview |
| Mattelin et al.  (2022) | Quantitative | 23.9 [SD 5.8] | 2.4% (4/164) had previous biological children  (also reports  2.1% (3/146) live births  0.7% (1/146) miscarriage  0.7% (1/146) termination of pregnancy) | Not reported | Not reported | Trans women 1.3% (1/78)  had previous biological children (as sperm donor) |
| Moseson et al. 2020, AND  Moseson et al. 2021 AND Moseson et al. 2022 | Quantitative and qualitative | 17 (median) range 18-78 | 10.7% ever pregnant (210/1964) (433 total pregnancies)  Results of 433 pregnancies:  Live birth (n=169, 39%),  Stillbirth 2 (0.5%)  Miscarriage (n=142, 33%)  Abortion (n=92, 21%).  Still pregnant (n=7, 2%)  Ectopic (n=2. 0.5%)  Unknown (n=19, 4%) | 3.4% ever had an abortion (67/1964)  Among 433 live births, 23% (n=39) were delivered via Caesarean section | Not reported | n/a |
| Obedin-Maliver et al. (2017) | Quantitative | 35.2 [SD 9.9] | 6.1% (2/33) parous  12.1% (4/33) gravid  3.0% (1/33) Caesarean delivery | Not reported | Not reported | 83.8 parous (712/850)  90.5% Gravid (769/850)  16.8% Caesarean delivery (143/850) |
| O’Hanlan et al. (2007) | Quantitative | 32.0 [SD NR] | 4.9% (2/41) parous | Not reported | Not reported | 62.9% parous (347/552) |
| Pfeffer et al.  (2023) AND Riggs et al. (2020) AND Riggs et al. (2021) | Qualitative and quantitative | 33 (mean age of the 51 who had been pregnant) | 72.8% (51/70) had been pregnant | 45 participants reported planned pregnancies, 6 reported unplanned pregnancies | Not reported | n/a |
| Reynolds et al.  (2021) | Quantitative | Not reported | 0.9% (7/800) transmasculine people had been pregnant  0.8% (17/2236) non-binary had been pregnant | 57.1% (4/7) unintended in trans students  82.3% (14/17) unintended in NB students | Not reported | 1.0% (1819/182,622) cisgender women had been pregnant |
| Riggs et al. (2016) | Quantitative | 39.8 [SD 13.5] | 42-63% (5/8 or 5/12) had given birth (unclear whether the gender diverse group of 4 had uteruses)  (3 gave birth before & 2 after transition) | Not reported | Not reported | 29 partners had given birth (19 before and 10 after transitioning) |
| Riggs & Bartholomaeus (2018) | Quantitative | 28.5 [SD 11.3] | 6.1% (8/131) men, 6.7% (10/149) NB^##^ participants reported having a previous pregnancy. | Not reported | Not reported | n/a |
| Stark et al. (2019) | Quantitative | 27.5 [SD 5.7] (range 21-50) | Ever pregnant 5.3% (8/150) range 1-3 pregnancies.  3.3% (5/150) had given birth (1-2 times)  20% (1/%) Caesarean section  80% (4/5) frontal delivery | Having social support associated with decreased odds of lifetime pregnancy (OR 0.83, 95% CI 0.68–0.99) | Not reported | n/a |
| Stroumsa et al.  (2023) | Quantitative | 23.48 [SD 5.50] – Medicaid  30 [SD 5.87] – commercial insurance | All had given birth  Medicaid cohort: Caesarean section 21% (54/256),  Commercial insurance cohort:  Caesarean section 22% (362/1651)  aOR Caesarean section (Medicaid) 0.47 (0.25–0.88), (commercial insurance) 0.55 (0.45–0.66) | Medicaid cohort –  Severe maternal morbidity % too few to calculate  Preterm birth 13% (33/256)  Commercial insurance cohort – Severe maternal morbidity 1.0% (16/1651)  Preterm birth 8% (133/1651)  aOR (adjusted for chronic and antenatal conditions)  Severe parental morbidity (Medicaid) 1.28 (0.29–5.57), (commercial insurance) 1.62 (0.98–2.69),  Preterm birth (Medicaid) 1.37 (0.94–2.01), (commercial insurance) 0.90 (0.75–1.09) | 40.2% (103/256) Black, 10.6% (27/256) Hispanic, 33.6% (86/256) White, < 11 other, (37/256 missing data) | Medicaid cohort –  Severe maternal morbidity 0.7% (9328/1255942)  Caesarean section, 28% (347677/1255942)  Preterm birth 11% (141290/1255942)  Commercial insurance cohort –  Severe maternal morbidity 0.7% (9844/1465565)  Caesarean section 34% (493548/1465565), Preterm birth 9% (125331/1465565) |
| Tordoff et al. (2019) | Quantitative | Not reported | 2% (5/131) trans men currently pregnant.  3% (5/98) GNC people currently pregnant. | Not reported | Not reported | 4% (2638/ 298,391) cis women currently pregnant.  Trans men and GNC people less likely to be pregnant than CW (trans PR=0.38, (95% CI 0.13-1.15) GNC (PR=0.76 (95% CI 0.23-2.58)) |
| Van Amesfoort et al.  (2023) | Qualitative | Range 23-35 | 100% (5/5) had previous pregnancy and live births  80% (4/5) had only 1 pregnancy  20% (1/5) had 3 pregnancies  80% (4/5) had 1 delivery  20% (1/5) had 2 deliveries  60% (3/5) vaginal births  40% (2/5) c sections | 20% (1/5) chest fed, 80% (4/5) did not chest feed | Not reported | n/a |
| Veale et al. (2016) | Quantitative | 20 [range 14-25] | Not reported | 0.2% (1/418) participants were currently pregnant. These 418 were aged 19-25 but proportion of AFAB not given | Not reported | n/a |
| Vyas et al.  (2021) | Quantitative | 38.5 [SD 16.8] | 8.3% (2/24^##^) AFAB participants had biological children | Not reported | Not reported | Not reported |
| Wierckx et al. (2012) | Quantitative | 37.0 [SD 8.2] | 6% (3/50) participants gave birth, (before hormonal or surgical interventions) | Quality of life for those with children significantly better than without for vitality and mental health, no significant differences for pain, general health, social, emotional or physical role or physical functioning | Not reported | n/a |
| Wingo et al. (2018) | Qualitative | 29.9 (range 18–44) | 7.7% (3/39) reported fertility assistance/pregnancy care | 5.1% (2/39) reported abortion access  10.3% (4/39) reported need for PCOS management.  5% (1/20) reported need for access to abortion care | 57% (22/39) White  15% (6/39) Black or African American  5% (2/39)Asian  8% (3/39) Biracial  15% (6/39)Hispanic | n/a |
| Yaish et al. (2021) | Quantitative | First sample 22.5 [IQR19–27.75]  Second sample: Median 24 [IQR 20-31] | 2.9% (3/103) had birthed children (conceived spontaneously after testosterone cessation and carried a pregnancy) | Not reported | Not reported | n/a |
| Yang et al.  (2023) | Quantitative | 30.8 [SD 5.98] | All were parents | 41.6% (96/231) trans men exclusively chestfed | Not reported | 28.3% (102/361) trans women,  29.1% (16/55) other exclusively chestfed |

\## - information from contacting the authors.

AFAB: assigned female at birth; aOR: adjusted odds ratio; CI: confidence intervals; GNC: gender non-conforming; IQR: interquartile range; IUI: intra-uterine insemination; IVF: in-vitro fertilisation; med: median; OR: odds ratio; NR: not reported; PR: prevalence ratio; SD: standard deviation; TNB: trans and non-binary;

**Table S4. Quality assessment using CASP questionnaire**

| Study | 1. Did the study address a clearly focussed issue? | 2. Did the authors use an appropriate method to answer their question | 3. Were the subjects recruited in an acceptable way? | 4. Were the measures accurately measured to reduce bias? | 5. Were the data collected in a way that addressed the research issue? | 6. Did the study have enough participants to minimise the play of chance? | 8. Was the data analysis sufficiently rigorous? | 9. Is there a clear statement of findings? | 10. Can the results be applied to the local population? | 11. How valuable is the research? |
| --- | --- | --- | --- | --- | --- | --- | --- | --- | --- | --- |
| Albar et al (2023) | Y | N | Y | Y | Y | N | Y | N | CT | CT |
| Baker et al. (2019) | Y | Y | CT | Y | Y | N | Y | Y | CT | CT |
| Brewer & Thomas (2019) | N | N | Y | N | CT | Y | N | N | CT | CT |
| Cao et al  (2021) | Y | Y | CT | Y | Y | N | Y | Y | CT | CT |
| Charlton et al  (2021) | Y | Y | CT | Y | Y | N | Y | Y | CT | CT |
| Cipres et al.  (2017) | Y | Y | Y | Y | Y | N | CT | Y | CT | CT |
| Copeland et al  (2023) | CT | N | CT | Y | Y | N | Y | N | CT | CT |
| Dantas  (2024) | Y | Y | CT | Y | Y | N | Y | Y | CT | CT |
| Ellis et al.  (2015) | Y | Y | CT | N | Y | N | Y | Y | CT | CT |
| Falck et al  (2021) AND Falck et al (2024) | Y | Y | CT | Y | Y | N | Y | Y | CT | CT |
| Fischer  (2021) | Y | Y | CT | Y | Y | N | Y | Y | CT | CT |
| Fix et al.  (2020) | N | N | CT | N | Y | N | CT | N | CT | CT |
| Galvao et al.  (2024) | Y | Y | CT | Y | Y | N | Y | Y | CT | CT |
| Ghofranian et al.  (2022) AND  Ghofranian  (2023) | Y | CT | CT | CT | CT | N | CT | N | CT | CT |
| Gomez et al.  (2020) | N | CT | CT | N | N | N | CT | N | CT | CT |
| Grimstad  (2023) | Y | Y | Y | Y | Y | N | Y | Y | CT | CT |
| Grimstad et al. (2019) | Y | Y | Y | Y | Y | N | Y | Y | CT | CT |
| Hawkins et al.  (2021) | Y | Y | Y | Y | Y | N | Y | Y | CT | CT |
| Jeftovic et al.  (2018) | Y | Y | Y | Y | Y | N | Y | Y | CT | CT |
| Leonard et al.  (2022) | Y | Y | Y | Y | Y | Y | Y | Y | Y | Y |
| Leung et al. (2019) | N | CT | CT | N | CT | N | N | N | CT | CT |
| Light et al. (2018) | Y | N | N | N | Y | N | N | CT | CT | CT |
| Light et al. (2014) | Y | Y | N | CT | Y | N | Y | Y | CT | CT |
| MacDonald et al. (2021) AND MacDonald et al. (2016) | Y | Y | CT | Y | Y | N | Y | Y | CT | CT |
| Maheux et al. (2021) | Y | Y | N | CT | Y | Y | CT | Y | CT | CT |
| Malmquist et al. (2021) | CT | N | CT | N | CT | N | N | CT | CT | CT |
| Mattelin et al.  (2022) | Y | Y | Y | Y | Y | N | Y | Y | CT | CT |
| Moseson et al.2020, AND  Moseson et al. 2021 AND Moseson 2022 | Y | CT | CT | CT | CT | N | N | Y | CT | CT |
| Obedin-Maliver et al. (2017) | Y | Y | Y | Y | Y | N | Y | Y | CT | CT |
| O’Hanlan et al. (2007) | Y | Y | Y | CT | Y | N | CT | CT | CT | CT |
| Pfeffer et al.  (2023) AND Riggs et al. (2020) AND Riggs et al. (2021) | Y | CT | CT | CT | CT | N | N | Y | CT | CT |
| Reynolds et al.  (2021) | Y | CT | CT | CT | CT | N | Y | Y | CT | CT |
| Riggs et al.  (2016) | CT | CT | CT | Y | Y | N | CT | Y | CT | CT |
| Riggs & Bartholomaeus (2018) | CT | N | CT | N | N | N | N | N | CT | CT |
| Stark et al.  (2019) | CT | CT | CT | N | CT | N | N | CT | CT | CT |
| Stroumsa et al.  (2023) | Y | CT | N | Y | Y | Y | Y | Y | CT | CT |
| Tordoff et al. (2019) | Y | Y | CT | Y | Y | Y | Y | Y | CT | CT |
| Van Amesfoort et al.  (2023) | Y | N | CT | Y | Y | N | Y | Y | CT | CT |
| Veale et al.  (2016) | N | CT | CT | CT | N | N | N | N | CT | CT |
| Vyas et al.  (2021) | N | CT | N | N | N | N | N | N | CT | CT |
| Wierckx et al. (2012) | Y | CT | N | Y | Y | N | N | Y | CT | CT |
| Wingo et al. (2018) | N | CT | CT | N | CT | N | CT | N | CT | CT |
| Yaish et al.  (2021) | N | N | CT | N | N | N | N | N | CT | CT |
| Yang et al.  (2023) | Y | N | CT | CT | CT | Y | CT | N | CT | CT |

**Table S5. Obstetric complications in participants with prior testosterone compared to none (from Light et al., 2014)**

|  | **Prior T (n=25)**  **n (%)** | **No prior T (n=16)**  **n (%)** |
| --- | --- | --- |
| Hypertension | 16% (4/25) | 6.3% (1/16) |
| Preterm labour | 12% (3/25) | 6.3% (1/16) |
| Placental abruption | 8% (2/25) | 12.5% (2/16) |
| Anaemia | 0 | 18.8% (3 /16) |
| Gestational diabetes | 8% (2/25) | 0 |
| Multiple pregnancy (twins) | 8% (2/25) | 0 |
| Postpartum infection | 4% (1/25) | 6.3% (1/16) |
| Premature rupture of membranes | 0 | 6.3% (1/16) |
| Pyelonephritis | 4% (1/25) | 0 |
| Uterine rupture | 4% (1/25) | 0 |
| Gestational age at delivery (wk, SD in days) | 37 (9) | 39 (5) |
| Caesarean section | 36% (9/25) | 18.8% (3/16) |
| Birth weight (g) (SD)¶ | 2,914 (SD 1,276) | 3,490 (SD 625) |
| Neonate admitted to the NICU | 16% (4/25) | 6.3% (1/16) |
| Chest (breast) fed | 40% (10/25)* | 68.8% (11/16)* |
| * p<0.05 | | |
